# PACE-NODES: A phase III randomised trial of 5 fraction prostate stereotactic body radiotherapy (SBRT) versus 5 fraction prostate and pelvic nodal SBRT

**DOI:** 10.1016/j.ctro.2026.101126

**Published:** 2026-02-11

**Authors:** Angela Pathmanathan, Suneil Jain, John Staffurth, Stephanie Brown, Stephanie Burnett, Fay H Cafferty, Ananya Choudhury, Monisha Dewan, Peter Hoskin, Ken McBride, Conor K McGarry, Elizabeth Miles, Julia Murray, Vedang Murthy, Olivia Naismith, Yee Pei Song, Isabel Syndikus, Alison Tree, Tim Ward, Emma Hall, Nicholas van As

**Affiliations:** a The Royal Marsden NHS Foundation Trust, London, UK; b Queen’s University, Belfast, UK; c Velindre University NHS Trust, UK; d Cardiff University, Cardiff, UK; e The Institute of Cancer Research, London, UK; f University of Manchester, Manchester, UK; g Belfast Health & Social Care Trust, UK; h Radiotherapy Trials QA Group (RTTQA), UK; i Mount Vernon Hospital, Northwood, UK; j The Christie NHS Foundation Trust, Manchester, UK; k The Clatterbridge Cancer Centre, Liverpool, UK; l NCRI Consumer Forum, London, UK; m Tata Memorial Centre, Mumbai, India; n Northern Ireland Cancer Research Consumer Forum, UK

**Keywords:** Prostate cancer, SBRT, Stereotactic body radiotherapy, Pelvic nodal radiotherapy, Randomised controlled trial

## Abstract

PACE-NODES is a phase III randomised controlled trial for patients with high risk localised prostate cancer, planned for radical radiotherapy with at least 12 months of androgen deprivation therapy, and suitable for stereotactic body radiotherapy (SBRT). Patients are randomised to receive either prostate SBRT (control) or prostate and pelvic nodal SBRT. In both arms, treatment is given in 5 fractions delivered on alternate days over 2 weeks. The aim of the trial is to determine whether prostate and pelvic nodal SBRT can improve outcomes for this patient group compared to prostate SBRT.

**Clinical trial registration:** PACE NODES is registered on ClinicalTrials.gov (NCT05613023).

## Highlights

• PACE-NODES is a randomised trial for high-risk localised prostate cancer patients.
• It will compare prostate and pelvic nodal SBRT with prostate-only SBRT.
• The primary endpoint is time to biochemical or clinical failure.
• Acute/late toxicity and patient-reported quality of life, will also be assessed.
• Recruitment is complete with primary analysis planned for 2029.

## 1 Introduction

In 2020, there were an estimated 1.4 million new prostate cancer cases and 375,000 deaths worldwide[1], [2]. Within the UK, the National Prostate Cancer Audit reports 14,560 of the 56,144 (26%) men diagnosed with prostate cancer in 2021 in England presented with high-risk localised or locally advanced disease[3]. Current clinical trials seek to improve the outcome of this group of patients with an emphasis on maximising the chance of cure, whilst minimising the risk of side-effects.

Older studies investigating prophylactic irradiation of the pelvic nodes have not shown a benefit[4], [5]. However, results are difficult to interpret due to patient selection, inconsistent staging and outdated radiotherapy techniques. With more conformal planning methods and image-guided radiotherapy (IGRT), dose escalation is feasible with limited increased dose to organs at risk, which may tip the balance in favour of nodal irradiation[6].

Several large, randomised trials, including the CHHiP (n = 3216) and PROFIT (n = 1206) trials have demonstrated that moderate hypofractionation delivering 60 Gy (Gy) at 3 Gy per fraction (f) achieves similar biochemical control compared to conventionally fractionated (2 Gy/f) treatment, without an increase in gastrointestinal (GI) or genitourinary (GU) toxicity[7], [8]. These have provided evidence for a low alpha–beta ratio and redefined the standard of care for radical treatment in localised prostate cancer. Trials of ultra-hypofractionated schedules, delivering doses of > 6 Gy/f, have reported outcomes similar to moderate hypofractionation albeit with a small and transient increase in moderate GU toxicity[9], [10], [11], [12], [13]. Ultra-hypofractionated schedules have additional benefits of reducing patient visits to hospital, limiting the burden on patients and radiotherapy departments, and reducing healthcare costs[14]. PACE-B (n = 874), a phase III randomised trial of prostate stereotactic body radiotherapy (SBRT) in men with low and intermediate risk prostate cancer, compared 5f SBRT at a dose of 36.25 Gy against standard conventional or moderately fractionated schedules (MHRT). Acute toxicity data confirms 5f SBRT is safe compared to standard radiotherapy[9]: in the standard and SBRT groups, the proportion of patients with acute RTOG ≥ grade 2 (G2+ ) GI toxicity was 12% and 10%, respectively and acute RTOG G2+ GU toxicity was 27% and 23%, respectively. These toxicity rates are lower than those seen in the CHHiP trial[15], likely due to mandated IGRT, smaller margins and more conformal planning techniques. In PACE-B, late toxicity demonstrates a higher cumulative incidence of G2+ GU toxicity (based on National Cancer Institute Common Terminology Criteria for Adverse Events, CTCAE) with SBRT compared with the standard of care group (32% vs 20%) and a similar cumulative incidence of G2+ CTCAE GI toxicity (12.5% vs 12.3%)[11]. Toxicity across both groups overall was low. The trial demonstrated non-inferiority of SBRT compared to standard of care in terms of biochemical and clinical failure-free survival up to 5-years post-treatment[12].

HYPO-RT-PC (n = 1200), which included 11% high-risk patients, compared conventionally fractionated treatment with extreme hypofractionated radiotherapy (EHFRT) delivering 6.1 Gy/f, demonstrating acceptable acute toxicity and quality of life (QoL)[10], [13]. Low rates of late toxicity were also reported with EHFRT (2-year cumulative incidence of RTOG Gr2+ GU and GI toxicity were 13% and 6% respectively, compared with 9% and 5% with conventional fractionation), and the trial demonstrated non-inferiority of EHFRT in terms of 5-year biochemical and clinical failure-free survival[13].

There is more limited data available for higher risk patients, with results awaited from the PACE-C trial [ISRCTN17627211, n = 1208] randomising higher risk patients (with up to 12 months androgen deprivation therapy (ADT)) to 20f prostate radiotherapy (MHRT) or 5f prostate SBRT (35% of patients high-risk). Assessing safety in this group is paramount to ensure that treatment remains safe and effective with more advanced disease and larger volumes being irradiated. Early data suggest a short-term increase in G2+ GI toxicity with SBRT that resolves by 12 weeks[16]. PACE-NODES seeks to demonstrate the efficacy and safety of 5f SBRT in an even higher risk group, with the inclusion of Gleason 9 and T3b/T4 stage disease.

Radiotherapy to the prostate and pelvic lymph nodes (PPN) gives acceptable toxicity but is normally given over several weeks, using a conventionally or moderately hypofractionated regime. The phase III PIVOTALboost trial [ISRCTN80146950, n = 2232] is comparing 20f PPN radiotherapy to prostate-only radiotherapy, with or without a boost to the dominant intraprostatic lesion[17]. The preceding multicentre phase II PIVOTAL study (n = 124) demonstrated PPN-IMRT (60 Gy over 7.5 weeks) is safe, with low additional toxicity compared to prostate radiotherapy[18]. A smaller, single centre phase I/II study has reported acceptable toxicity rates with PPN-IMRT given over four weeks, with cumulative 2-year RTOG G2+ GI and GU toxicity of 16% and 5% respectively[6]. The randomised POP-RT trial (n = 224) reported improved outcome with the addition of whole pelvic radiotherapy, with a 5-year biochemical failure-free survival of 95% versus 81% for prostate-only radiotherapy (HR 0.23, 95% CI: 0.10 to 0.52, p = 0.0001) using 25f[19]. Patients were eligible for this study with a risk of nodal involvement of at least 20%, using the Roach formula[20], with 47% of patients having at least T3b disease. This is the first randomised evidence to demonstrate a benefit for pelvic nodal radiation and, of relevance, approximately 80% of patients underwent PSMA-PET-CT imaging for staging.

With the movement towards extreme hypofractionation, several smaller, early phase trials have treated high-risk prostate cancer with PPN-SBRT, including FASTR, FASTR-2, SATURN and PRIME[21], [22], [23], [24]. The SPORT trial [NCT03253978] is a single-centre study randomising 30 patients with unfavourable intermediate- and high-risk localised prostate cancer to prostate-only SBRT 36.25 Gy in 5f, or PPN-SBRT, with additional 25 Gy to the pelvic nodes[25]. This demonstrated that PPN-RT in five visits was technically feasible with acceptable side effects. Data from a systematic review and from the SHARP consortium confirms the acceptable toxicity profile and excellent pelvic nodal control with this dose[26], [27]. We now need to prospectively test the tumour control impact of PPN-SBRT in high-risk prostate cancer at multiple centres. With this preliminary data on pelvic nodal SBRT, the encouraging results for nodal radiotherapy from POP-RT in the 25f setting and UK trials of both 20f PPN-RT and 5f prostate SBRT anticipated to report efficacy results within the next 5 years, PACE-NODES is the natural next step.

## 2 Design

**Fig. 1:**
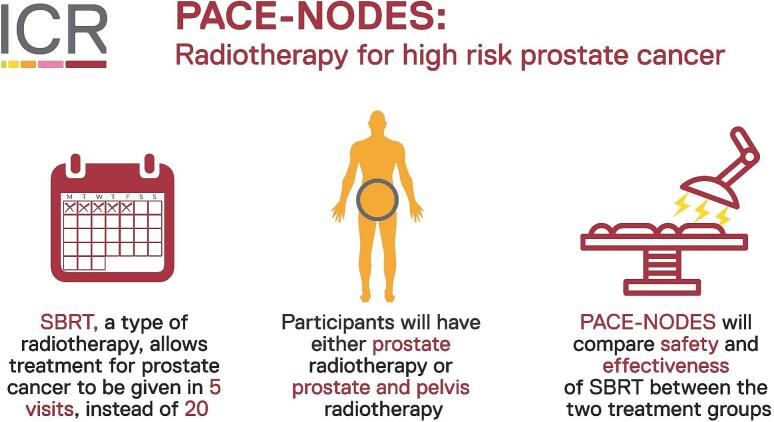
PACE-NODES graphical summary.

**Fig. 2:**
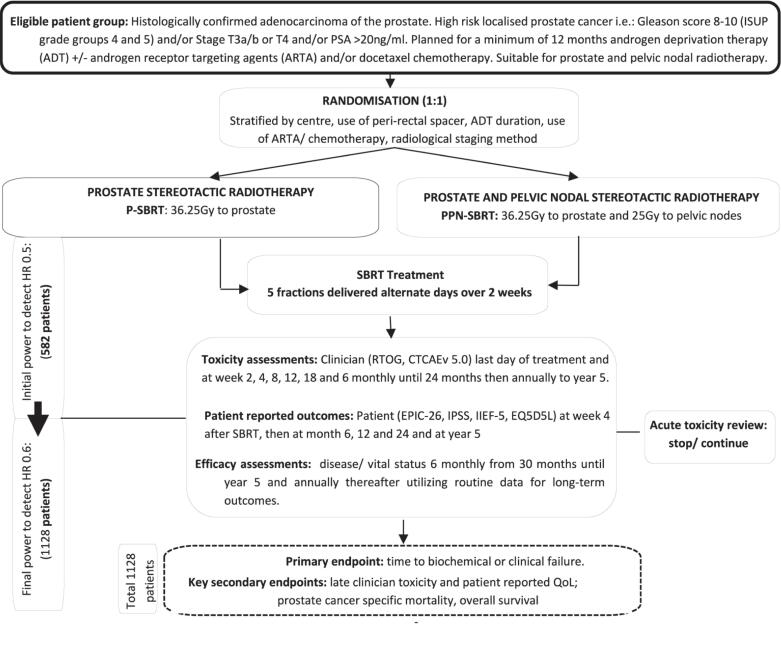
PACE-NODES trial schema and randomisation.

PACE-NODES is a multicentre, phase III, randomised trial with the primary aim of determining superiority of PPN-SBRT over prostate-only SBRT, in terms of time to biochemical or clinical failure (Fig. 1). The trial opened in September 2022 and recruited patients with histologically confirmed prostate cancer that is localised and high-risk, requiring a minimum of 12 months of ADT. Participants are randomised 1:1 to prostate and seminal vesicles SBRT (P-SBRT) or prostate, seminal vesicles and pelvic node SBRT (PPN-SBRT), both in 5-fractions on alternate days (Fig. 2). The full protocol is included as appendix A.

### 2.1 Study objectives

The primary objective is to determine whether 5-fraction PPN-SBRT has superior biochemical/clinical-failure free rate (reduces the risk of biochemical or clinical failure by 40% or more) than 5-fraction P-SBRT, in patients with high-risk localised prostate cancer.

Secondary objectives are:

• To assess acute and late GI and GU toxicity with P-SBRT and PPN-SBRT.
• To rule out a doubling in the cumulative rate of late GI toxicity (occurring 6–24 months after treatment) for PPN-SBRT compared to P-SBRT.
• To assess patient-reported QoL in relation to bowel/urinary effects and late erectile dysfunction for P-SBRT and PPN-SBRT.
• To assess efficacy of the two treatment approaches in terms of subsequent occurrence of metastatic disease and (prostate cancer) deaths.
• To demonstrate feasibility with respect to radiotherapy planning and delivery (adherence to pre-specified dose constraints) in a multicentre setting.

### 2.2 Eligibility

Inclusion criteria for the trial are as follows:

• Histopathological confirmation of prostate adenocarcinoma within 12 months of randomisation
• Planned for 12–36 months ADT
• High-risk localised prostate cancer, defined by:
- Gleason 8–10 (grade groups 4 and 5) and/or
- Stage T3a/b or T4 and/or
- PSA > 20 ng/ml
• Bi- or multi-parametric MRI of the pelvis − to include at least one functional MRI sequence in addition to T2W imaging ideally within 12 months prior to starting ADT
• Radiological staging to exclude metastatic disease, ideally within a maximum of 3 months prior to starting ADT
• WHO Performance status 0–2

Exclusion criteria are:

• N1 or M1 disease
• PSA > 50 ng/ml unless PET-CT imaging has confirmed N0M0 disease
• Previous active treatment for prostate cancer
• Patients where SBRT is contraindicated − prior pelvic radiotherapy, inflammatory bowel disease, significant lower urinary tract symptoms
• Patients in whom diagnostic MRI has shown bowel in close apposition to target volumes that would make pelvic radiotherapy highly unlikely to be deliverable
• Contraindications to fiducial marker insertion
• Bilateral hip prostheses or any other implants introducing substantial imaging artefacts
• Patients who have had chemotherapy within 6 weeks of starting radiotherapy.
• Life expectancy < 5 years

### 2.3 Treatment, allocation and assessments

#### 2.3.1 Oncological treatments

All participants will receive ADT for 12–36 months as part of standard care. Use of androgen receptor targeting agents (ARTA) and/or up to 6 cycles of docetaxel is permitted, if part of standard of care, but not mandated.

Patients are allocated 1:1 to P-SBRT or PPN-SBRT using minimisation with a random element. Balancing factors are: centre, radiological staging method (PET-CT and/or whole body MRI; other), use of *peri*-rectal spacers, planned duration of ADT and use of ARTA/chemotherapy. Treatments are delivered in 5-fractions on alternate days as follows:

P-SBRT: Prostate and seminal vesicles SBRT, receiving 36.25 Gy in 5-fractions (40 Gy to prostate clinical target volume (CTV)) on alternate days. This regimen is similar to the common experimental arm of the PACE trial platform (PACE-A, PACE-B and PACE-C)[9].

PPN-SBRT: Prostate, seminal vesicles and pelvic node SBRT, receiving 36.25 Gy to the prostate (40 Gy to prostate CTV) and 25 Gy to the pelvic nodes.

##### 2.2.3.2 Radiotherapy quality Assurance (RTQA)

A comprehensive QA programme for the PACE-NODES trial has been designed and implemented by the National Institute of Health Research Radiotherapy Trials Quality Assurance (NIHR RTTQA) group, including pre-trial and on-trial components. The QA processes for PACE-NODES have been streamlined for centres that have already completed the QA programme for other prostate trials, for example PIVOTALboost, PEARLS [ISRCTN36344989] and PACE.

For pre-trial QA, centres must complete the following prior to site activation: 1) facility questionnaire, 2) benchmark outlining cases, and 3) benchmark planning cases. On-trial QA includes prospective and/or retrospective case review, dosimetry site visit (dependent on prior RTQA dosimetry accreditation) and Digital Imaging and Communications in Medicine data collection for all patients.

Radiotherapy planning and delivery guidelines are provided as appendix B.

##### 2.2.3.3 Trial assessment

The schedule of assessments and follow-up is shown in Table 1. All participants are asked to provide consent for collection of diagnostic prostate biopsy samples for future research.

**Table 1:** Schedule of Assessments.

**Visit/Assessment**	**Pre-randomisation (for eligibility)**	**Pre-treatment**	**Last fraction of SBRT**	**Follow-up post completion of treatment**								
				Week 2	Week 4	Week 8	Week 12	Month 6	Month 12	Month 18	Month 24	Year 3–5
Medical history and concomitant medication check	X											
PSA^1^	X							X	X	X	X	X^6^
Testosterone												X^2^
MRI pelvis^3^	X											
Additional imaging^4^	X											
CTCAE		X	X	X	X	X	X	X	X	X	X	X
RTOG: bladder and bowel		X	X	X	X	X	X	X	X	X	X	X
QOL: IPSS		X			X			X	X		X	X^5^
QOL: EPIC-26		X			X			X	X		X	X^5^
QOL: EQ-5D-5 L		X			X			X	X		X	X^5^
QOL: IIEF-5		X										X^5^

1. Pre-ADT PSA at pre-randomisation; 2. Testosterone at year 5/month 60 only; 3. MRI is mandatory pre-biopsy for staging purposes; 4. Radiological staging to exclude metastatic disease with one of the following- PSMA PET-CT, fluciclovine/choline PET-CT, whole-body MRI, bone scan, CT of chest, abdomen and pelvis (imaging method as per local practice/standard of care). Where available, PET-CT is strongly advised. *N.B. PSA > 50 ng/ml (or > 25 ng/ml for patients on 5-alpha reductase inhibitors), unless PET-CT imaging has been performed to confirm N0M0 diseas*e.; 5. QOL questionnaires to be completed at month 60/year 5 only; 6. PSA every 6 months during years 3–5.

Serious Adverse Events (SAEs) are defined as any untoward medical occurrence that occurs after the first study intervention and within 30 days of the last treatment administration.

### 2.4 Endpoints

The primary outcome is freedom from biochemical or clinical failure. Biochemical (PSA) failure follows the RTOG-ASTRO *Phoenix* Consensus definition requiring an increase in serum PSA ≥ 2 ng/ml greater than the post-treatment nadir[28]. Clinical failure is defined as local recurrence, lymph node/pelvic recurrence, distant metastases, recommencement of ADT or death due to prostate cancer. This includes recurrence identified on PSMA-PET or whole-body MRI.

Evaluation of gastrointestinal toxicity is particularly relevant for PPN-SBRT given the increased bowel dose compared to P-SBRT. As such, a key secondary endpoint is late G2+ GI toxicity (CTCAE v5.0) occurring at any point between 6–24-month follow-up.

Other secondary endpoints include:

• Time to recommencement of ADT; biochemical failure; loco-regional recurrence
• Metastasis-free survival
• Prostate cancer-specific survival
• Overall survival
• Acute (within 12 weeks of treatment) and late (from 6 months post-treatment) toxicity, both measured with CTCAE and RTOG
• Patient-reported QoL measured using: the International Prostate Symptom Score (IPSS), the 5-item version of the International Index of Erectile Function (IIEF-5), the short form Expanded Prostate Index Composite-26 (EPIC-26) and the EQ-5D-5L
• Adherence to organ at risk doses constraints
• Second primary cancer incidence

### 2.5 Statistics

#### 2.2.5.1 Sample size for primary efficacy analysis – Initial trial design

The original expectation for the 5-year event-free rate (where an event is biochemical or clinical failure) in the P-SBRT control arm was 81% based on the POP-RT trial[19] and anticipated event rates for trials in lower-risk groups, i.e. PACE and PIVOTALboost[11], [15]. The target hazard ratio (HR) for the trial was initially 0.5, corresponding to a 9% improvement (to 90%) in the 5-year event-free rate with PPN-SBRT. Although a larger effect (HR = 0.23) was seen in POP-RT, the PACE-NODES design aims to ensure that a smaller but clinically meaningful effect is not missed whilst also reflecting a magnitude of target effect size which is sufficiently large to counterbalance a possible increase in toxicity. The original target effect size was slightly larger than for PIVOTALboost (HR = 0.625), which is testing the benefit of adjuvant pelvic nodal radiotherapy in the setting of 20f radiotherapy and a lower risk group of patients. A total of 70 events (46 in the P-SBRT arm, 24 in the PPN-SBRT arm) were required to achieve 80% power using a 5% two-sided significance level. Assuming a 2-year recruitment period with analysis taking place after a minimum of 3.5 years follow-up and 3% loss to follow-up, the original target sample size was 536 patients[6].

#### 2.2.5.2 Changes to the sample size for primary efficacy analysis

Ahead of reaching the original recruitment target, PACE-B results were presented showing excellent outcomes for both standard prostate RT and prostate SBRT. This led the PACE-NODES Trial Management Group to review the event rate assumptions that had been used for PACE-NODES and, as a result, the target sample size was increased to 1128 (protocol version 3.0, April 2024). This reflected two changes: (i) an increase in the expected event-free rate in the control arm (from 81% to 85% at 5 years) based on data from PACE-B suggesting an improvement in outcomes over time[29]; and (ii) a more conservative target effect size (changing the HR from 0.5 to 0.6, equating to an absolute increase of 6% in the 5-year event-free rate with PPN-SBRT) to ensure that a smaller, but still clinically meaningful, difference could be detected. This latter change followed the observation that use of PSMA-PET for staging within PACE-NODES was lower than in POP-RT, with the potential to dilute any observed benefit of PPN-SBRT. In order to achieve 80% power (with 5% two-sided significance level), 126 events are required (78 in the P-SBRT arm, 48 in the PPN-SBRT arm) and, after adjusting timelines and expected rates of accrual based on recruitment up to that point, this gave a required sample size of 1128.

#### 2.2.5.3 Power for secondary toxicity analysis

This number of patients also gives adequate power for secondary toxicity comparisons, aimed at demonstrating non-inferiority of PPN-SBRT in terms of late GI toxicity (CTCAE Gr2+ ). We assumed a 92% evaluable rate (based on expected event-free rate at 2-years and loss to follow-up), estimating that there would be 1038 patients evaluable for toxicity comparison up to 2 years. Assuming a toxicity rate of 13% with P-SBRT (based on PACE-B), the trial will have more than 85% power, with a one-sided 5% significance level, to rule out a PPN-SBRT toxicity rate of > 19%, i.e. accepting an increase of up to 6% worse[9].

#### 2.2.5.4 Interim analyses and stopping rules

The trial was designed to be a single stage, phase III trial with no interim efficacy analyses planned. However, emerging safety and efficacy data are reviewed at least annually by an Independent Data Monitoring Committee (IDMC).

Two pre-specified interim safety analyses were planned. Radiotherapy delivery and dose constraints data, as well as acute toxicity, for the first 15 patients randomised to PPN-SBRT were to be reviewed to confirm the feasibility of the radiotherapy protocol and that treatment is being delivered within safe constraints without significant target undercoverage. Then, acute toxicity data would be reviewed once 82 patients in the PPN-SBRT arm had been treated and followed up for 12 weeks. Results were to be reviewed by the IDMC who will make recommendations regarding continuation and/or modification of the trial based on the following pre-specified guidelines:

i. Acute CTCAE G2+ GU symptoms were reported in 31% of patients in the PACE-B SBRT arm by 12 weeks [9]; if G2+ GU symptoms are reported in 37 or more (≥45%) of the 82 PPN-SBRT patients, this would be cause for concern. A 90% confidence interval would not be able to exclude a rate of 55%.
ii. Acute CTCAE G2+ GI symptoms were reported in 16% of patients in the PACE-B SBRT arm by 12 weeks [9]; if G2+ GI symptoms are reported in 25 or more (≥30%) of the 82 PPN-SBRT patients, this would be cause for concern. A 90% confidence interval would not be able to exclude a rate of 40%.

Both of these reviews have taken place and the IDMC recommended continuation of the trial with no changes.

## 3 Planned timeline

PACE-NODES recruited its first patient on 9th September 2022 and is open in 40 UK centres, 3 Irish centres and 1 New Zealand centre. The planned recruitment timeline was initially 2.5 years, extended to 3 years with the increase in sample size. Recruitment was completed ahead of target in July 2025. The primary efficacy analysis is planned for 2029.

## 4 Discussion

PACE-NODES addresses a unique question on the benefit of pelvic nodal irradiation in men with high-risk localised prostate cancer receiving SBRT to the prostate. It will provide comparative data on the effect of pelvic nodal SBRT on acute and late toxicity and any impact on patients’ QoL. PACE-NODES complements the ProtecT, CHHiP, PIVOTALboost and PACE trials, and the currently recruiting PEARLS trial[8], [12], [17], [30], [31]. The trial also provides opportunities for translational research, through collection of diagnostic prostate biopsy samples from > 99% recruited patients who have consented to donate tissue. This resource will complement other sample collections, including those associated with the PIVOTALBoost and PEARLS trials (via the 3P sample collection programme; CRCPSC-Jul23/100002) also studying “volume” questions in this setting. An exceptional rate of recruitment has allowed key modifications to be made to the sample size to reflect latest evidence and ensure an appropriate effect size is targeted. This highlights the opportunities for conducting and successfully delivering radiotherapy trials in the UK, especially for men with prostate cancer.

## CRediT authorship contribution statement

**Angela Pathmanathan:** Conceptualization, Funding acquisition, Investigation, Methodology, Writing – review & editing. **Suneil Jain:** Conceptualization, Funding acquisition, Investigation, Methodology, Writing – original draft, Writing – review & editing. **John Staffurth:** Conceptualization, Funding acquisition, Investigation, Methodology, Writing – original draft, Writing – review & editing. **Stephanie Brown:** Data curation, Project administration, Writing – review & editing. **Stephanie Burnett:** Data curation, Methodology, Project administration, Writing – review & editing. **Fay H Cafferty:** Data curation, Formal analysis, Methodology, Project administration, Resources, Writing – original draft, Writing – review & editing. **Ananya Choudhury:** Conceptualization, Funding acquisition, Investigation, Methodology, Writing – review & editing. **Monisha Dewan:** Data curation, Methodology, Writing – review & editing. **Peter Hoskin:** Investigation, Methodology, Writing – review & editing. **Ken McBride:** Writing – review & editing. **Conor K McGarry:** Writing – review & editing. **Elizabeth Miles:** Project administration, Methodology, Writing – review & editing. **Julia Murray:** Investigation, Methodology, Writing – review & editing. **Vedang Murthy:** Writing – review & editing. **Olivia Naismith:** Data curation, Methodology, Writing – review & editing. **Yee Pei Song:** Investigation, Methodology, Writing – review & editing. **Isabel Syndikus:** Investigation, Methodology, Writing – review & editing. **Alison Tree:** Funding acquisition, Investigation, Methodology, Writing – review & editing. **Tim Ward:** Conceptualization, Methodology, Writing – review & editing. **Emma Hall:** Conceptualization, Data curation, Formal analysis, Funding acquisition, Methodology, Project administration, Resources, Writing – review & editing. **Nicholas van As:** Conceptualization, Funding acquisition, Investigation, Methodology, Writing – review & editing.

## Ethics approval

PACE-NODES was approved by the London − Chelsea Research Ethics Committee (REC reference 22/LO/0263) and by the relevant Institutional Review Boards of sites participating in Ireland and New Zealand.

## Funding

PACE NODES is funded by Prostate Cancer UK (PCUK grant number MA-CT20-005).

The trial is sponsored by The Institute of Cancer Research and centrally managed by the Cancer Research UK-funded Institute of Cancer Research Clinical Trials and Statistics Unit (ICR-CTSU, grant reference C1491/A25351). Radiotherapy quality assurance was provided by the National Institute for Health and Care Research (NIHR) funded National Radiotherapy Trials Quality Assurance Group. PACE-NODES was made possible in Ireland due to sponsorship/coordination by Cancer Trials Ireland and funding support from Irish Cancer Society (ICS) and Health Research Board (HRB). PACE-NODES is funded in New Zealand by the Auckland Medical Research Foundation.

## Declaration of competing interest

The authors declare that they have no known competing financial interests or personal relationships that could have appeared to influence the work reported in this paper.
