# Supplementary material for: PACE-NODES: A phase III randomised trial of 5 fraction prostate stereotactic body radiotherapy (SBRT) versus 5 fraction prostate and pelvic nodal SBRT
Source: Clin Transl Radiat Oncol. 2026 Feb 11;58:101126. doi: 10.1016/j.ctro.2026.101126 (PMC12925429; doi:10.1016/j.ctro.2026.101126)
Supplement: Supplementary Data 1 [file mmc1.pdf]

PACE-NODES

A phase III randomised trial of 5 fraction prostate SBRT versus 5 fraction prostate and pelvic nodal SBRT

PROTOCOL

Version: 3
Dated: 17th April 2024

Chief Investigator:	Professor Nicholas van As
Co-Clinical Leads:	Dr Angela Pathmanathan and Professor Suneil Jain
Sponsor:	The Institute of Cancer Research
Funder:	Prostate Cancer UK (PCUK)
Coordinating Trials Unit:	ICR Clinical Trials and Statistics Unit (ICR-CTSU)<br>The Institute of Cancer Research

REC Reference Number:	22/LO/0263307888
IRAS Reference Number:	307888
ICR-CTSU Protocol Number:	ICR-CTSU/2021/10079
Sponsor Number:	CCR5545
ClinicalTrials.gov Identifier	NCT05613023
NIHR Reference Number:	52562

The PACE-NODES study is part of the National Institute for Health Research Clinical Research Network Trial Portfolio

CONFIDENTIAL

This protocol is a controlled document and should not be copied, distributed, or reproduced without the written permission of the ICR-CTSU

ADMINISTRATION

Clinical Coordination

Professor Nicholas van As (Chief Investigator)
Tel: 0207 811 8127
Nicholas.vanAs@rmh.nhs.uk

Dr Angela Pathmanathan (Clinical Lead)
Tel: 0208 661 3271
Angela.Pathmanathan@icr.ac.uk

Professor Suneil Jain (Clinical Lead)
Tel: 028 9504 8549
s.jain@qub.ac.uk

Trial Coordination

ICR-CTSU (a CRUK funded, UKCRC Registered Clinical Trials Unit (CTU) and member of the NCRI Cancer CTU Group) is responsible for the central coordination of the trial on behalf of the Sponsor.

ICR Clinical Trials & Statistics Unit (ICR-CTSU),
Division of Clinical Studies,
The Institute of Cancer Research,
Sir Richard Doll Building,
Cotswold Road,
Sutton, Surrey SM2 5NG

ICR-CTSU Methodology Lead:
Emma Hall
Tel: 020 8722 4013
Emma.Hall@icr.ac.uk

ICR-CTSU Principal Statistician:
Fay Cafferty
Tel: 020 3437 6794
Fay.Cafferty@icr.ac.uk

ICR-CTSU Clinical Trials Programme Manager:
Stephanie Burnett
Tel: 020 8722 4261
Stephanie.Burnett@icr.ac.uk

ICR-CTSU Statistician:
Monisha Dewan
Tel: 020 8722 4008
Monisha.Dewan@icr.ac.uk

PACE-NODES Senior Trial Manager:
Stephanie Brown
Tel: 020 8722 4467
PACE-NODES-icrctsu@icr.ac.uk

Radiotherapy Trials QA Physicist:
Olivia Naismith
Tel: 020 7808 2500
Olivia.Naismith@rmh.nhs.uk

Any questions relating to this protocol should be addressed in the first instance to the PACE-NODES Clinical Trial Manager within ICR-CTSU:

Email: PACE-NODES-icrctsu@icr.ac.uk
General enquiries: 020 8722 4467

PROTOCOL DEVELOPMENT GROUP

Nicholas van As	Chief Investigator	The Royal Marsden NHS Foundation Trust
Emma Hall	Deputy Director (Research)	ICR-CTSU, The Institute of Cancer Research, London
Angela Pathmanathan	Clinical Lead	The Royal Marsden NHS Foundation Trust
Suneil Jain	Clinical Lead	Queen's University, Belfast
Ananya Choudhury	Chair and Honorary Consultant in Clinical Oncology	University of Manchester
John Staffurth	Professor in Clinical Oncology	Cardiff University
Alison Tree	Consultant Clinical Oncologist	The Royal Marsden NHS Foundation Trust
Julia Murray	Consultant Clinical Oncologist	The Royal Marsden NHS Foundation Trust
Peter Hoskin	Consultant Clinical Oncologist	University of Manchester
Conor McGarry	Medical Physicist	Belfast Health & Social Care Trust
Elizabeth Miles	National RTTQA Group Lead	Mount Vernon Cancer Centre
Olivia Naismith	Radiotherapy Trials Physicist	The Royal Marsden NHS Foundation Trust
Yee Pei Song	Consultant Clinical Oncologist	The Christie NHS Foundation Trust
Isabel Syndikus	Consultant Clinical Oncologist	The Clatterbridge Cancer Centre
Tim Ward	Patient Representative	
Stephanie Burnett	Clinical Trials Programme Manager	ICR-CTSU, The Institute of Cancer Research, London
Fay Cafferty	Principal Statistician	ICR-CTSU, The Institute of Cancer Research, London

Protocol Authorised by:

Name & Role	Signature	Date
Nicholas van As<br>(Chief Investigator)	[signature]	17 April 2024
Emma Hall<br>(ICR-CTSU Methodology Lead)	[signature]	17 April 2024

This protocol describes the PACE-NODES trial and provides information about procedures for entering participants into this trial. The protocol should not be used as a guide for the treatment of patients outside of this trial.

Every care was taken in the preparation of this protocol, but corrections or amendments may be necessary. Protocol amendments will be circulated to participating sites as they occur, but sites entering patients for the first time are advised to contact ICR-CTSU to confirm they have the most recent version.

HISTORY OF PROTOCOL AMENDMENTS

PROTOCOL VERSION AND DATE	SUMMARY OF CHANGES
1.0	Original protocol
2.0	Addition of SPRUCE study within a trial<br>Addition of second primary cancer as a secondary endpoint<br>Addition of ADT duration </≥ 2years as an additional balancing factor<br>Removal of requirement for consecutive PSA rises to allow for ‘PSA bounce’<br>Imaging timing requirements changed to relate to ADT start rather than randomisation
3.0	Sample size increased from 536 to 1128.<br>Expected event free rate and effect size reviewed with the effect of increasing sample size.<br>Site numbers and international site participation details updated.<br>Strengthened wording around exclusion of patients where the diagnostic MRI showed bowel in close apposition to target volumes.<br>Confirmation that consent can only be taken by a qualified clinician.<br>Randomisation procedure updated to reflect current practice.<br>Lymphedema added as an expected adverse event.

CONTENTS

1. INTRODUCTION ... 1

1.1. Background ... 1
1.2. Extreme hypofractionation ... 1
1.3. Pelvic nodal radiotherapy ... 2
1.4. Description of Population ... 2
1.5. Study Rationale ... 2

2. TRIAL OBJECTIVES ... 3

2.1. Primary Objective ... 3
2.2. Secondary Objectives ... 3

3. TRIAL DESIGN ... 3
4. STUDY ENDPOINTS ... 4

4.1. Primary Endpoint ... 4
4.2. Secondary Endpoints ... 4

5. PATIENT SELECTION & ELIGIBILITY ... 4

5.1. Number of Participants ... 4
5.2. Source of Participants ... 4
5.3. Inclusion Criteria ... 4
5.4. Exclusion Criteria ... 5
5.5. Life Style Guidelines ... 5

6. SCREENING ... 5

6.1. Screening Log ... 5
6.2. Procedure for Obtaining Informed Consent ... 6
6.3. Participation in other Clinical Trials ... 6

7. RANDOMISATION ... 7
8. TRIAL ASSESSMENTS ... 8

8.1. Pre-Randomisation Evaluations (required for eligibility) ... 8
8.2. Pre-treatment Assessments ... 8
8.3. Evaluation during and following treatment ... 9
8.4. Discontinuation from Treatment ... 9
8.5. Discontinuation from Follow-up ... 10
8.6. Schedule of Assessments ... 11

9. TRIAL TREATMENT ... 12

9.1. Randomisation options ... 12
9.2. Treatment timelines ... 12
9.3. Systemic treatment ... 12
9.4. Treatment technique ... 12
9.5. Radiotherapy Planning ... 13
9.6. Treatment Scheduling and Gaps ... 13
9.7. Concomitant Therapy ... 13
9.8. Non-permissible Medications/Therapies ... 13

10. Radiotherapy Quality Assurance (QA) ... 14
11. Safety Reporting ... 14

11.1. Definitions ... 14
11.2. Reporting Adverse Events to ICR-CTSU ... 15

11.3. Reporting of Serious Adverse Events to ICR-CTSU 16
11.4. Review of Serious Adverse Events 16
11.5. Expedited Reporting of Related Unexpected SAEs 16
11.6. Follow up of Serious Adverse Events 17
11.7. Annual Safety Reporting 17
11.8. Reporting Pregnancies 17

12. STATISTICAL CONSIDERATIONS 19

12.1. Statistical Design and Sample Size Justification 19

12.1.1. Sample size for primary efficacy analysis 19
12.1.2. Power for secondary toxicity analysis 20

12.2. Treatment Allocation 20
12.3. Endpoint Definitions 20

12.3.1. Primary endpoint 20
12.3.2. Secondary endpoints 21

12.4. Statistical Analysis Plan 22
12.5. Interim Analyses and Stopping Rules 23

13. TRIAL MANAGEMENT 24

13.1. Trial Management Group (TMG) 24
13.2. Trial Steering Committee (TSC) 24
13.3. Independent Data Monitoring Committee (IDMC) 24

14. RESEARCH GOVERNANCE 25

14.1. Sponsor Responsibilities 25
14.2. Participating Site Responsibilities 25

15. TRIAL ADMINISTRATION & LOGISTICS 25

15.1. Site Activation 25
15.2. Data Acquisition 25
15.3. Central Data Monitoring 25
15.4. On-Site Monitoring 26
15.5. Completion of the Study and Definition of Study End Date 26
15.6. Archiving 26

16. PATIENT PROTECTION AND ETHICAL CONSIDERATIONS 26

16.1. Risk Assessment and Approval 26
16.2. Public and Patient Involvement 26
16.3. Ethics Approvals 26
16.4. Trial Conduct 27
16.5. Informed Consent 27
16.6. Patient Confidentiality 27
16.7. Data Protection 27
16.8. Liability 28

17. FINANCIAL MATTERS 28
18. PUBLICATION POLICY 28
19. ASSOCIATED STUDIES 28

19.1. Translational Study 28
19.2. Quality of Life Study 29

20. REFERENCES 30

A1. WHO performance status ... 32
A2. RTOG Toxicity Scales ... 33
A3. Expected serious adverse events ... 35
A4. GLOSSARY ... 36

PACE-NODES TRIAL SUMMARY

PROTOCOL TITLE	**PACE-NODES:** A phase III randomised trial of 5 fraction prostate SBRT (P-SBRT) versus 5 fraction prostate and pelvic nodal SBRT (PPN-SBRT)
TARGET DISEASE	1. Histopathological confirmation of prostate adenocarcinoma with Gleason/ISUP grade group scoring<br>2. Patients planned for of 12-36 months androgen deprivation therapy<br>3. High risk localised prostate cancer as defined by<br>- Gleason 8-10 (grade groups 4 and 5) and/or<br>- Stage T3a/b or T4 and/or<br>- PSA > 20ng/ml<br>4. Multi-parametric/bi-parametric MRI of the pelvis- to include at least one functional MRI sequence in addition to T2W imaging, ideally no more than twelve months before starting ADT.<br>5. Radiological staging to exclude metastatic disease (ideally within 3 months prior to starting ADT) with one of the following- PSMA PET-CT, fluciclovine/choline PET-CT, whole-body MRI, bone scan, CT of chest, abdomen and pelvis (imaging method as per local practice/standard of care).
TRIAL OBJECTIVES	The primary objective is to determine whether PPN-SBRT has superior biochemical/clinical-failure free rate than P-SBRT, in patients with high risk localised prostate cancer.
TRIAL DESIGN	PACE-NODES is a multicentre phase III randomised controlled trial
TRIAL POPULATION	Patients with high risk localised prostate cancer, deemed suitable for SBRT radiotherapy and planned for 12 - 36 months androgen deprivation therapy
RECRUITMENT TARGET	The aim is to recruit 1128 participants; 564 into each arm of the study
TRIAL TREATMENT	Patients will be allocated to one of two treatment arms:<br>1. Prostate alone SBRT (P-SBRT) to receive 36.25Gy in 5 fractions to the prostate and seminal vesicles<br>2. Prostate and pelvic node SBRT (PPN-SBRT) to receive 36.25Gy in 5 fractions to the prostate and seminal vesicles and 25Gy in 5 fractions to pelvic nodes
PRIMARY ENDPOINT	Time to biochemical or clinical failure as defined by time from randomisation to the first biochemical failure, local recurrence,

	lymph node/pelvic recurrence, distant metastases, recommencement of androgen deprivation therapy or death due to prostate cancer
SECONDARY ENDPOINTS	1. Clinical reported acute and late toxicity using CTCAE version 5.0 and RTOG criteria. Focus will be given to GU and GI Grade 2 or higher (G2+) toxicities.<br>2. Metastatic relapse-free survival, prostate cancer-specific survival and overall survival<br>3. PROMs as assessed by IPSS, EPIC-26, EQ-5D and IIEF-5<br>4. Adherence to radiotherapy protocol<br>5. Incidence of second primary cancers
FOLLOW UP	Evaluation during and after treatment will be as follows;<br>1. Acute toxicity will be assessed at the final fraction, then 2, 4, 8 and 12 weeks after end of treatment<br>2. Late toxicity will be assessed at 6, 12, 18, 24, 36, 48 and 60 months.<br>3. Quality of life questionnaires will be completed at 4 weeks after treatment and then at 6, 12, 24 and 60 months after the end of treatment.<br>4. PSA (for determination of biochemical failure) will be collected at 6 months following completion of SBRT, then 6-monthly for 5 years.<br>Long-term data capture will be pursued through routine data sources.

TRIAL SCHEMA

PACE-NODES is a multi-centre, randomised phase III trial which recruits men with high risk localised prostate cancer to receive either 5 fraction prostate SBRT (P-SBRT) or prostate and pelvic node SBRT (PPN-SBRT).

Figure 1: Trial Schema

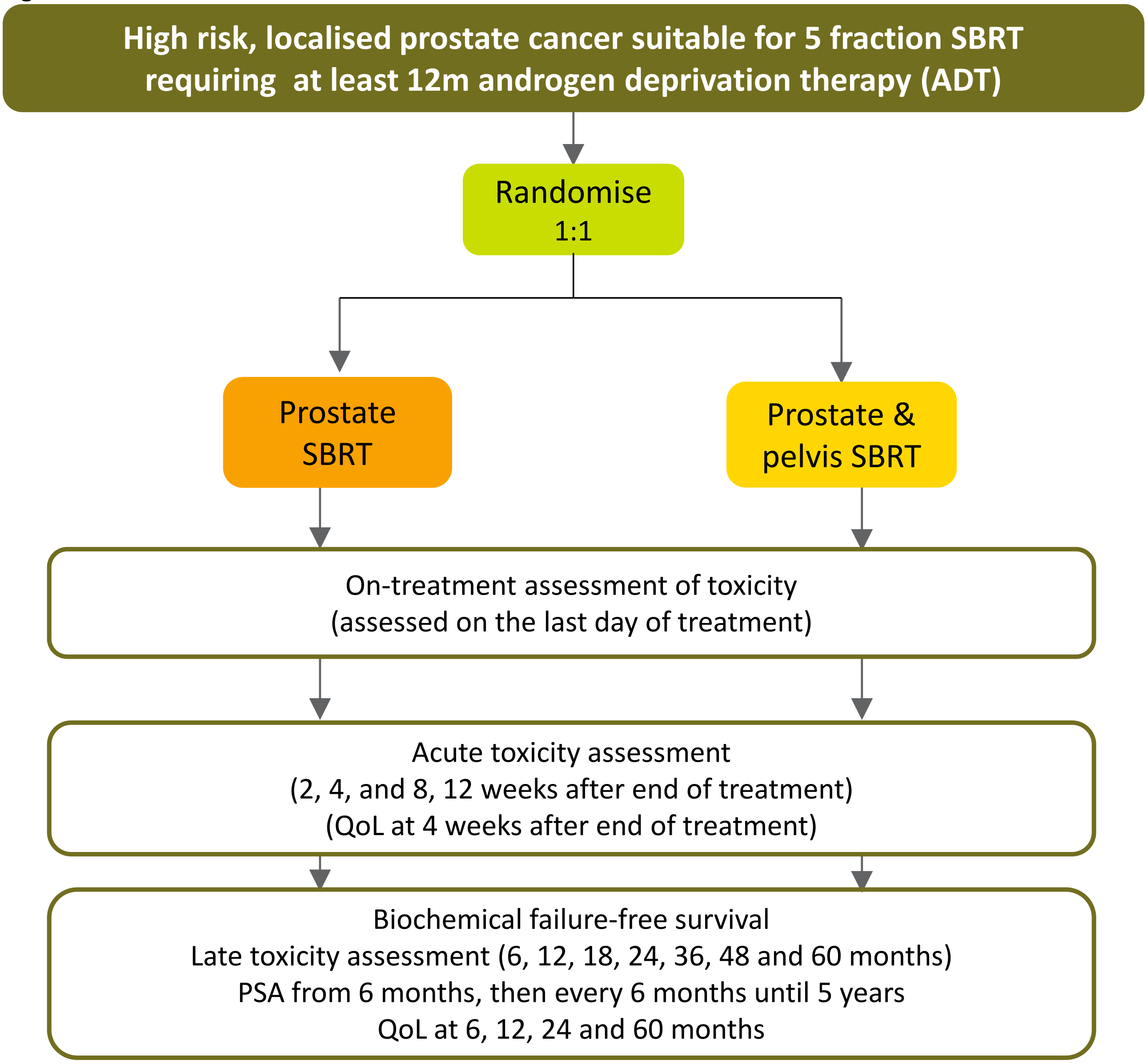

- Toxicity will be assessed using Radiation Therapy Oncology Group (RTOG) grading and the National Cancer Institute Common Terminology Criteria for Adverse Events (CTCAE) version 5.0.
- Patient reported International Prostate Symptom Score (IPSS), Expanded Prostate Index Composite-26 (EPIC-26) and EuroQol-5D (EQ-5D) QOL will be measured at baseline, 4 weeks after treatment and then at 6, 12, 24 and 60 months from the end of radiotherapy. The International Index of Erectile Function 5 (IIEF-5) will be measured at 60 months.
- All participants will be followed up for at least 3.5 years with trial specific follow-up to 5 years.

1. INTRODUCTION

1.1. Background

Despite the increasing sensitivity of imaging, the presence of undiscovered micrometastatic disease in prostate cancer patients with a higher risk of pelvic nodal involvement [1] increases the risk of recurrence, in particular in patients with high-risk disease. Current clinical trials seek to improve the outcome of this group of patients with an emphasis on maximising the chance of cure, whilst minimising the risk of side effects.

Older trials investigating the prophylactic irradiation of the pelvic nodes have not shown a benefit [2, 3]. However, results are difficult to interpret due to patient selection, inconsistent staging to rule out metastatic disease and outdated radiotherapy techniques. With more conformal delivery methods and image-guided radiotherapy (IGRT), safer dose escalation allows the dose to the organs at risk to be minimised, which may tip the balance in favour of nodal irradiation.

1.2. Extreme hypofractionation

With increasing evidence for a low alpha-beta ratio for prostate cancer [4], several large randomised trials, including the CHHiP trial, have demonstrated that moderate hypofractionation achieves similar biochemical control compared to conventional treatment, without an increase in gastrointestinal (GI) or genitourinary (GU) toxicity [5-7]. These have redefined standard of care for radical treatment in localised prostate cancer.

Extreme hypofractionated schedules, delivering doses of >6 Gray (Gy) per fraction (f), have similar side-effects to moderate hypofractionation and may improve outcome [1, 8]. They have additional benefits of being more patient-friendly and reducing the burden on radiotherapy departments, with an associated reduction in healthcare costs [9].

PACE-B, a phase III randomised clinical trial of prostate stereotactic body radiotherapy (SBRT) in men with low and intermediate risk prostate cancer, compared 5 fraction SBRT at a dose of 36.25Gy against standard conventional or moderately fractionated schedules. Acute toxicity data confirms 5-fraction SBRT is safe compared to the current 20 fraction standard of care [1]. In the standard and SBRT groups, the proportion of patients with acute RTOG $\geq$ grade 2 GI toxicity was 12% and 10% respectively and acute RTOG $\geq$ grade 2 GU toxicity was 27% and 23% respectively. These toxicity rates are lower than those seen in the CHHiP trial [3], likely to be due to a combination of mandated IGRT, smaller margins and more conformal planning techniques within the PACE-B trial. Recent late toxicity data from PACE-B demonstrates a higher cumulative incidence (to 27 months) of Gr2+ CTCAE GU toxicity with SBRT compared with the standard of care group (32.2% vs 19.8%), and a similar cumulative incidence of Gr2+ CTCAE GI toxicity (12.5% vs 12.3%) [10]. Toxicity across both groups overall was low. HYPO-RT-PC, which also included 11% high risk patients, compared conventional/moderately fractionated treatment with extreme hypofractionated radiotherapy (EHFRT) schedules delivering >6Gy/f, demonstrating acceptable acute toxicity [1, 8]. Low rates of late toxicity were also reported with EHFRT (2-year cumulative incidence of RTOG Gr2+ GU and GI toxicity were 13% and 6% respectively, compared with 9% and 5% with conventional fractionation), and the trial demonstrated non-inferiority of EHFRT compared to conventionally fractionated treatment in terms of biochemical and clinical failure-free survival up to 5 years post-treatment.

There is more limited data available for higher risk patients, with the ongoing PACE-C trial randomising higher risk patients (up to 12 months ADT) to 20 fraction prostate radiotherapy or 5 fraction prostate SBRT.

Assessing safety in this group is paramount to ensure that treatment remains safe and effective with more advanced disease and larger volume seminal vesicle inclusion. However, PACE-NODES seeks to demonstrate the efficacy and safety in an even higher risk group, with the inclusion of Gleason 9 and T3b/T4 staged disease.

1.3. Pelvic nodal radiotherapy

Radiotherapy to the prostate and lymph nodes gives acceptable toxicity but is normally given over several weeks, using a moderately hypofractionated regime. The phase III PIVOTALboost trial is comparing 20 fraction prostate and pelvis radiotherapy to prostate alone, with or without a boost to the dominant intraprostatic lesion. The preceding multi-centre phase II PIVOTAL study demonstrated intensity modulated radiotherapy (IMRT) to the prostate and pelvic nodes (60Gy over 7.5 weeks) is safe with low additional toxicity compared to prostate radiotherapy [5]. A smaller single centre phase I/II study has reported acceptable toxicity rates with pelvic nodal radiotherapy given over four weeks, with cumulative 2 year RTOG > grade 2 GI and GU toxicity of 16.4% and 4.8% respectively [7].

In January 2021, the 224 patient phase III randomised POP-RT trial [10] reported improved outcome with the addition of whole pelvic radiotherapy, with a 5 year biochemical failure-free survival of 95.0% versus 81.2% for prostate-only radiotherapy giving a hazard ratio of 0.23 for biochemical failure (95% CI: 0.10 to 0.52, p=0.0001) using 25 fractions. The hazard ratio for disease-free survival was 0.4 (95% CI, 0.22 to 0.73, p=0.002). Patients were eligible for this study with a risk of nodal involvement of at least 20%, as determined using the Roach formula [11] with 46.8% of patients having at least T3b disease. This is the first randomised evidence to demonstrate a benefit for pelvic nodal radiation and of relevance, approximately 80% of patients underwent PSMA PET CT imaging for staging.

With the movement towards extreme hypofractionation, several smaller, early phase trials have treated high risk prostate cancer with prostate and pelvic nodal SBRT, including FASTR [12], FASTR-2 [13], SATURN [14] and PRIME [15]. The SPORT trial (NCT03253978) is a single-centre study randomising 30 patients with unfavourable intermediate and high risk localised prostate cancer to prostate alone 36.25Gy in 5f, or prostate and pelvic node SBRT, with additional 25Gy to the pelvic nodes. This demonstrated that radiotherapy to the prostate and lymph glands in five visits was technically feasible with acceptable side effects [16]. We now need to test SBRT to the prostate and pelvic lymph nodes in high risk prostate cancer at multiple centres.

With this preliminary data on pelvic nodal SBRT, the encouraging results for nodal radiotherapy from POP-RT in the 25 fraction setting and UK trials of both 20 fraction prostate and pelvic lymph node radiotherapy and 5 fraction prostate SBRT anticipated to report efficacy results within the next 5 years, PACE-NODES is the natural next step.

1.4. Description of Population

Patients with newly diagnosed high risk localised prostate cancer, deemed suitable for SBRT and planned for a minimum of 12 months androgen deprivation therapy.

1.5. Study Rationale

With increasing evidence for prostate SBRT in low and intermediate risk localised prostate cancer, and the increased risk of micrometastatic nodal disease in men with high risk localised prostate cancer, PACE-NODES

seeks to assess the efficacy and safety of 5 fraction SBRT to the prostate, with or without pelvic nodes, in this group of patients.

2. TRIAL OBJECTIVES

2.1. Primary Objective

To determine whether 5 fraction prostate and pelvic node SBRT (PPN-SBRT) has a superior biochemical/clinical-failure free rate (reduces the risk of biochemical of clinical failure by 40% or more) than 5 fraction prostate SBRT (P-SBRT), in patients with high risk localised prostate cancer.

2.2. Secondary Objectives

The trial has the following secondary objectives:

- to assess acute and late GI and GU toxicity with SBRT and PPN-SBRT; and, specifically, to rule out a doubling in the cumulative rate of late gastrointestinal (GI) toxicity (occurring between 6 and 24 months after treatment) with PPN-SBRT when compared to P-SBRT.
- to assess patient reported outcome measures (PROMs) of bowel, urinary and late erectile dysfunction with the two treatments.
- to assess efficacy of the two treatment approaches in terms of subsequent occurrence of metastatic disease and (prostate cancer) deaths.
- to demonstrate feasibility of PPN-SBRT with respect to radiotherapy planning and delivery (adherence to pre-specified dose constraints) in a multi-centre setting.

3. TRIAL DESIGN

PACE-NODES is a multicentre phase III randomised trial with the primary aim of determining superiority of PPN-SBRT over P-SBRT, in terms of time to biochemical or clinical failure.

Patients enrolled in this study will have high risk localised prostate cancer, as specified in the inclusion criteria, requiring a minimum of 12 months of androgen deprivation therapy (ADT) and will be randomised to one of two groups:

- **Prostate and seminal vesicles SBRT (P-SBRT)**
    - 36.25Gy in 5 fractions on alternate days (as used in the common experimental arm of the PACE trial platform (PACE-A, PACE-B and PACE-C)).

- **Prostate, seminal vesicles and pelvic node SBRT (PPN-SBRT)**
    - 36.25Gy in 5 fractions to the prostate and 25Gy in 5 fractions to the pelvic nodes on alternate days

4. STUDY ENDPOINTS

4.1. Primary Endpoint

The primary endpoint is time to biochemical or clinical failure.

- Biochemical (PSA) failure is defined as time from randomisation to the first biochemical failure, following the RTOG-ASTRO Phoenix Consensus definition of an increase in serum PSA ≥2ng/ml greater than the post-treatment nadir [17].
- Clinical failure is defined as time from randomisation to first local recurrence, lymph node/pelvic recurrence, distant metastases, recommencement of androgen deprivation therapy or death due to prostate cancer. This includes recurrence identified on PSMA PET or wbMRI.

4.2. Secondary Endpoints

- Clinical reported acute and late toxicity using CTCAE version 5.0 and RTOG criteria. Focus will be given to GU and GI Grade 2 or higher (G2+) toxicities.
- Metastatic relapse-free survival, prostate cancer-specific survival, and overall survival
- Time to recommencement of androgen deprivation therapy; biochemical failure; loco-regional recurrence (recurrence in the prostate and/or pelvic lymph nodes)
- PROMs as assessed by IPSS, EPIC-26, EQ-5D and IIEF-5
- Adherence to radiotherapy protocol
- Incidence of second primary cancers

5. PATIENT SELECTION & ELIGIBILITY

5.1. Number of Participants

The aim is to recruit 1128 participants; 564 into each group of the study.

5.2. Source of Participants

Participants will be recruited from approximately 40 participating sites in the UK, and additional international centre participation from Ireland and New Zealand.. Potential participants will be identified in oncology clinics and discussed at Multi-Disciplinary Team (MDT) meetings.

ICR-CTSU encourages investigators to consider equality, diversity and inclusion when recruiting participants into its trials.

5.3. Inclusion Criteria

1. Aged ≥ 18 years at randomisation.
2. Histopathological confirmation of prostate adenocarcinoma with Gleason/ISUP grade group scoring within twelve months of randomisation (unless otherwise discussed with the CI or co-Clinical Leads).
3. Patients planned for 12-36 months androgen deprivation therapy.

4. High risk localised prostate cancer as defined by
   - Gleason 8-10 (grade groups 4 and 5) and/or
   - Stage T3a/b or T4 and/or
   - PSA > 20ng/ml (or >10 ng/ml for patients on 5-alpha reductase inhibitors)
5. Multi-parametric/bi-parametric MRI of the pelvis- to include at least one functional MRI sequence in addition to T2W imaging (ideally no more than 12 months before starting ADT).
6. Radiological staging to exclude metastatic disease, (ideally within 3 months prior to starting ADT), with one of the following: PSMA PET-CT, fluciclovine/choline PET-CT, whole-body MRI, bone scan, CT of chest, abdomen and pelvis (imaging method as per local practice/standard of care).
7. WHO performance status 0-2.
8. Ability of research subject to give written informed consent.

5.4. Exclusion Criteria

1. N1 or M1 disease
2. PSA >50ng/ml (or >25ng/ml for patients on 5-alpha reductase inhibitors), unless PET-CT imaging has been performed to confirm N0M0 disease .
3. Previous active treatment for prostate cancer.
4. Patients where SBRT is contraindicated: prior pelvic radiotherapy, inflammatory bowel disease, significant lower urinary tract symptoms.
5. Patients in whom diagnostic MRI has shown bowel in close apposition to target volumes that would make pelvic radiotherapy highly unlikely to be deliverable should be excluded.
6. Contraindications to fiducial marker insertion, where used- including clotting disorders, or patients at high risk when stopping anticoagulation or antiplatelet medications.
7. Bilateral hip prostheses or any other implants/hardware that would introduce substantial CT artefacts and would make pelvic node planning more difficult.
8. Patients who have had chemotherapy within 6 weeks of the start of radiotherapy.
9. Life expectancy < 5 years, whether due to a previous malignancy or other significant medical co-morbidity.

5.5. Life Style Guidelines

It is unlikely that the patient population included in PACE-NODES will be at risk of fathering a child. However, if this is a possibility for any individual patient, this and sperm banking should be discussed, and the patient should be advised to use effective contraception and avoid conception for 12 months after treatment.

Effective contraception is defined as double barrier contraception (e.g. condom plus spermicide in combination with a diaphragm, cervical cap or intrauterine device).

6. SCREENING

6.1. Screening Log

We may request participating sites to keep a log of all participants with high risk localised prostate cancer that are potentially eligible for this study. The information collected on the log will include:

- Date patient identified
- Screening outcome (patient approached/accepted participation/declined participation)
- Reasons for not approaching / declining participation (if available)
- Trial ID (if applicable)

This information may be used by the TMG to monitor recruitment activity. No patient identifiable data will be sent to ICR-CTSU at this stage.

6.2. Procedure for Obtaining Informed Consent

The Principal Investigator (PI) or suitably trained designated clinician as delegated by the local PI, must ensure that each trial patient is fully informed about the nature and objectives of the trial and possible risks associated with participation. Participants should be given the current ethics approved PACE-NODES patient information sheet (PIS) for their consideration. A large print PIS and an audio PIS to aid blind/partially sighted patients will be available upon request. Patients should only be asked to consent to the study after they have had sufficient time to consider the trial, and the opportunity to ask any further questions. This consent process may take place remotely where appropriate, such as during a telephone or video consultation, providing the terms for informed consent as outlined have been met.

No protocol required assessments should be conducted until the PACE-NODES consent form has been signed and dated by both the patient and the Investigator, unless they are performed routinely as part of standard patient care.

All participants will be asked for consent to collect diagnostic prostate biopsy samples (FFPE blocks) for future research. Refusal to consent to the tissue collection will not result in ineligibility to participate in the main clinical trial and will not impact the medical care received.

Confirmation of the patient's consent and the informed consent process must be documented in the patient's medical notes. A copy of the signed consent form should be provided to the patient and the original retained in the investigator site file, which must be available for verification by ICR-CTSU study staff or for regulatory inspection at any time.

6.3. Participation in other Clinical Trials

Patients who fulfil the eligibility criteria will be given the opportunity to participate in PACE-NODES if they have participated in other clinical trials prior to recruitment.

Participation in other interventional clinical trials will be considered on a case-by-case basis by the Trial Management Group.

Participation in the ICR-CTSU study within a trial investigating electronic collection of patient reported outcomes, SPRUCE, is permitted.

7. RANDOMISATION

Patients must have their treatment allocated centrally by ICR-CTSU before trial treatment can commence.

Patients should be randomised by emailing ICR-CTSU on:
Randomisation-icrctsu@icr.ac.uk

The randomisation email account is monitored 09.00-17.00 (UK time) Monday to Friday*

** New Zealand patients should be randomised by emailing pace-nodes-icrctsu@icr.ac.uk*

Treatment allocation should take place as close to the planned start date of treatment as possible. An eligibility and randomisation checklist must be completed prior to treatment allocation.

The following information will be required at treatment allocation:

- Name of hospital, consultant and person registering patient
- Confirmation that patient has given written informed consent for trial and for any sub-studies;
- Confirmation that patient is eligible for the trial by completion of the eligibility checklist
- Patient's full name#, hospital number#, date of birth (partial date of birth for international participants), postcode# and NHS/CHI number#
- Use of peri-rectal spacers for radiotherapy treatment (yes/no)
- Prior use of docetaxel and/or use of androgen receptor targeting agents (yes/no)

# Not required for international sites

The patient will be assigned a unique randomisation number (Trial ID) and treatment allocation will be confirmed.

ICR-CTSU will send confirmation via email to delegated individuals at the recruiting site to confirm a patient's entry into the trial.

8. TRIAL ASSESSMENTS

Patient will be screened for eligibility based on the inclusion/exclusion criteria.

8.1. Pre-Randomisation Evaluations (required for eligibility)

The following assessments should be conducted prior to randomisation:

- Medical history and concomitant medication check (within 6 weeks preceding randomisation).
- Pathological confirmation of adenocarcinoma of the prostate with Gleason scoring within 12 months of randomisation (unless otherwise discussed with the CI or co-Clinical Leads).
- PSA for eligibility to be checked prior to starting ADT.
- WHO performance status (within 6 weeks preceding randomisation).
- Multiparametric/bi-parametric MRI of the pelvis prior to biopsy for staging purposes (ideally no more than 12 months before starting ADT), to include at least one functional sequence in addition to T2-weighted imaging.
- Radiological staging to exclude metastatic disease (ideally within 3 months prior to starting ADT) with one of the following- PSMA PET-CT, fluciclovine/choline PET-CT, whole-body MRI, bone scan, CT of chest, abdomen and pelvis (imaging method as per local practice/standard of care). Where available, PET-CT is **strongly recommended as standard of care**. *N.B. PSA >50ng/ml (or >25ng/ml for patients on 5-alpha reductase inhibitors), unless PET-CT imaging has been performed to confirm N0M0 disease.*

8.2. Pre-treatment Assessments

The following assessments should be conducted:

- Baseline symptoms, assessed using Common Toxicity Criteria for Adverse Event Reporting (CTCAE) version 5 and RTOG bladder and bowel toxicity scoring. This should be done after consent and ideally **before fiducial insertion** where used, whilst the patient is on ADT.
- If they have consented to the QoL sub study, patients should complete the baseline booklet of quality of life patient questionnaires:
    - International Prostate Symptom Score (IPSS)
    - The Expanded Prostate Index Composite-26 (EPIC-26) short form questionnaire
    - EuroQol 5 dimensions and 5 levels (EQ-5D-5L) questionnaire
    - International Index for Erectile Function-5 (IIEF-5)

Where participating sites are taking part in the SPRUCE study (REC Ref: 21/WM/0223) PACE-NODES participants should be approached about SPRUCE and enrolled following completion of the PACE-NODES baseline booklet and randomisation into PACE-NODES. All PACE-NODES follow up quality of life questionnaires will be administered directly to participants by ICR-CTSU for patients participating in SPRUCE.

8.3. Evaluation during and following treatment

Patients will be assessed at the completion of radiotherapy and following treatment, as per the table in section 8.9.

At all time points, toxicity assessment will record the maximal toxicity since the last toxicity assessment.

Follow up may be performed as a telephone or video consultation, at the discretion of the treating clinician.

- Participants will be assessed for acute toxicity at 2, 4, 8 and 12 weeks after end of treatment.
- Thereafter participants will be reviewed for late toxicity at 6, 12, 18, 24, 36, 48 and 60 months.
- For participants that have consented, quality of life questionnaires will be completed at 4 weeks after treatment and then at 6, 12, 24 and 60 months following the end of treatment.
- If a patient relapses, it is assumed that imaging at relapse mirrors imaging pre-trial entry in line with local practice/standard of care.

PSA (for determination of biochemical failure) will be collected following the end of treatment at 6 months, then 6-monthly up to 5 years.

Every effort should be made to ensure that the questionnaires (which may be administered on paper or electronically) are completed. Where hard copy booklets are used, please aim to ensure that all questions and all pages have been completed by the patient when the booklet is handed in. From 6-months onwards questionnaires will be administered by ICR-CTSU directly to the patient*. See section 19.2 for full details regarding administration of the quality of life booklets.

**For international sites all quality of life booklets will be administered by the site*

Post treatment assessments should take place within the following visit windows:

- End of treatment: ± 3 days
- Week 2 and Week 4 visit: ± 3 days
- Week 8 and Week 12 visit: ± 1 week
- Month 6: ± 2 weeks
- Month 12 and thereafter: ± 4 weeks

Long-term data capture will be pursued through routine data sources.

8.4. Discontinuation from Treatment

Participants may discontinue from trial treatment at any time at their own request, or they may be discontinued at the discretion of the Principal Investigator. Reasons for discontinuation may include:

- Disease progression or recurrence
- Unacceptable toxicity

Participants who discontinue treatment should continue to be followed up unless the participant explicitly withdraws their consent for further follow-up (see section 8.5 below).

8.5. Discontinuation from Follow-up

If a patient withdraws from further follow-up a trial deviation form should be submitted to ICR-CTSU stating whether the patient simply no longer wishes to attend trial follow up visits or whether the patient has withdrawn consent for any further information to be sent to the ICR-CTSU.

8.6. Schedule of Assessments

**Visit/Assessment**	**Pre-randomisation (for eligibility)**	**Pre-treatment**	**Last frxn of SBRT**	**Follow-up post completion of treatment**								
				Week 2	Week 4	Week 8	Week 12	Month 6	Month 12	Month 18	Month 24	Year 3-5
Medical history and concomitant medication check	X											
PSA[1]	X							X	X	X	X	X[6]
Testosterone												X[2]
MRI pelvis[3]	X											
Additional imaging[4]	X											
CTCAE		X	X	X	X	X	X	X	X	X	X	X
RTOG: bladder and bowel		X	X	X	X	X	X	X	X	X	X	X
QOL: IPSS		X			X			X	X		X	X[5]
QOL: EPIC-26		X			X			X	X		X	X[5]
QOL: EQ-5D-5L		X			X			X	X		X	X[5]
QOL: IIEF-5		X										X[5]

1. Pre-ADT PSA at pre-randomisation; 2. Testosterone at year 5/month 60 only; 3. MRI is mandatory pre-biopsy for staging purposes; 4. Radiological staging to exclude metastatic disease with one of the following- PSMA PET-CT, fluciclovine/choline PET-CT, whole-body MRI, bone scan, CT of chest, abdomen and pelvis (imaging method as per local practice/standard of care). Where available, PET-CT is strongly advised. *N.B. PSA >50ng/ml (or >25ng/ml for patients on 5-alpha reductase inhibitors), unless PET-CT imaging has been performed to confirm N0M0 disease*.; 5. QOL questionnaires to be completed at month 60/year 5 only; 6. PSA every 6 months during years 3-5.

9. TRIAL TREATMENT

9.1. Randomisation options

Patients will receive one of two treatment protocols:

1) Participants allocated P-SBRT will receive:
   - 36.25Gy in 5 fractions to the prostate and seminal vesicles (40Gy to prostate clinical target volume (CTV)).
2) Participants allocated PPN-SBRT will receive:
   - 36.25Gy in 5 fractions to the prostate and seminal vesicles (40Gy to prostate CTV)
   - 25Gy in 5 fractions to pelvic nodes.

All participants will receive long course androgen deprivation therapy (ADT), for 12 to 36 months, as part of standard care.

9.2. Treatment timelines

There is no restriction on length of time on ADT prior to randomisation. However, radiotherapy treatment should ideally commence within 6 months of starting hormone therapy, and no more than 12 months from starting ADT.

9.3. Systemic treatment

All participants will receive long course ADT, for between 12 and 36 months, as part of standard care. Planned duration of ADT will be collected at entry to the trial and used as a balancing factor in the minimisation algorithm. Drugs will be provided by standard local arrangements. Treatment may include luteinising hormone-releasing hormone (LHRH) agonist, LHRH antagonist, bicalutamide monotherapy or combined androgen blockade at the clinician's discretion.

Androgen receptor target agents (ARTA) such as Enzalutamide, Abiraterone or Apalutamide are permitted and included as an additional minimisation balancing factor.

Docetaxel chemotherapy will be permitted, where a minimum of 9 weeks washout period is present between day 1 of the final cycle of chemotherapy and the start of SBRT (and included as an additional minimisation balancing factor, together with ARTA).

9.4. Treatment technique

All radiotherapy techniques are to be approved in advance by the Chief Investigator and/or the co-clinical leads and the PACE trial radiotherapy QA team.

- Image-guided radiotherapy (IGRT) is mandatory for all patients, with fiducial marker based IGRT **strongly recommended**. Fiducial markers are not necessary for MRI guided radiotherapy.
- Treatment will be delivered on alternate days in both treatment arms.

9.5. Radiotherapy Planning

Radiotherapy planning and outlining should be carried out in accordance with the guidelines in the current version of the radiotherapy planning document, available on request from ICR-CTSU (PACE-NODES-icrctsu@icr.ac.uk) and via the RTTQA website (http://www.rttrialsqa.org.uk/rttqa/).

- Radiotherapy will be planned according to randomisation allocation, using pre-specified planning constraints for the target volumes and organs at risk. In patients where mandatory constraints are not met, cases will be discussed on an individual basis with the trial clinical and RTTQA team.
- If there are planning issues in relation to SBRT for pelvic nodes for patients randomised to the PPN-SBRT arm and the treatment cannot be delivered, alternative treatment options should be discussed with the Chief Investigator and/or Clinical Coordinators and the RTTQA team. Please see the RTQA guidance notes for further information.
- Dose constraints will be based on those from the PACE-C trial, with additional constraints for PPN-SBRT from the SPORT trial. Bowel constraints are stricter than previously reported studies in order to prioritise bowel tolerance.
- Peri-rectal spacers are permitted as part of the treatment for patients where this will not compromise target coverage or toxicity, and must not be used in patients with posterior T3a/T4 disease. In centres using peri-rectal spacers, this must be used for patients randomised to either arm i.e. the decision to use a peri-rectal spacer must be made prior to randomisation.

9.6. Treatment Scheduling and Gaps

Treatment can start on any day of the week. Treatment should be given in a single phase and should ideally be planned to be delivered within 14 days of starting.

9.7. Concomitant Therapy

All medication considered necessary for the patients' welfare, and which is not expected to interfere with the evaluation of the treatment may be given at the discretion of the investigator. All concomitant medications (including start/stop dates, dose frequency, route of administration and indication), must be recorded in the patient's notes, as well as the appropriate pages of the CRF.

9.8. Non-permissible Medications/Therapies

Non-permissible concurrent medications/therapies include:

- Chemotherapy (including chemotherapy for concurrent non-prostate cancers)

10. Radiotherapy Quality Assurance (QA)

A quality assurance program will be instigated to ensure the safety and consistency of radiotherapy delivery at participating sites. For further information please see the Radiotherapy Quality Assurance guidance notes.

11. Safety Reporting

11.1. Definitions

Adverse Event (AE)
An AE is any untoward medical occurrence in a patient administered a study treatment; the event does not necessarily have a causal relationship with the treatment.

Serious Adverse Event (SAE)
An SAE is any untoward medical occurrence that occurs after the first study intervention and within 30 days of the last treatment administration and:

- results in death,
- is life-threatening
- requires hospitalisation or prolongation of existing inpatients´ hospitalisation
- results in persistent or significant disability or incapacity
- is a congenital anomaly or birth defect

Important adverse events that are not immediately life-threatening or do not result in death or hospitalisation but may jeopardise the subject or may require intervention to prevent one of the other outcomes listed in the definition above, may also be considered serious.

Progression of the indicated disease and death due to progression of the indicated disease are not considered SAEs.

Pregnancy or aid in the conception of a child whilst participating in a trial is not itself considered an SAE but should be followed up for congenital anomalies or birth defects.

Serious Adverse Reaction (SAR)
A serious adverse reaction is an SAE that is suspected as having a causal relationship to the trial treatment (SBRT), as assessed by the investigator responsible for the care of the patient. A suspected causal relationship is defined as possibly, probably or definitely related (see definitions of causality table).

Definitions of causality

Relationship	Description
Unrelated	There is no evidence of any causal relationship with the trial treatment
Unlikely	There is little evidence to suggest there is a causal relationship (e.g. the event did not occur within a reasonable time after administration of the trial treatment). There is another reasonable explanation for the event (e.g. the patient's clinical condition, other concomitant treatment)
Possible	There is some evidence to suggest a causal relationship (e.g. because the event occurs within a reasonable time after administration of the trial treatment). However, the influence of other factors may have contributed to the event (e.g. the patient's clinical condition, other concomitant treatments)
Probable	There is evidence to suggest a causal relationship, and the influence of other factors is unlikely
Definitely	There is clear evidence to suggest a causal relationship, and other possible contributing factors can be ruled out
Not assessable	There is insufficient or incomplete evidence to make a clinical judgement of the causal relationship.

Related **Unexpected Serious Adverse** Event
An adverse event that meets the definition of serious and is assessed by the CI or nominative representative as:

- "Related" – that is, it resulted from administration of any of the research procedures, and
- "Unexpected" – that is, the type of event is not listed in the protocol as an expected occurrence (see appendix 3)

11.2. Reporting Adverse Events to ICR-CTSU

Any toxicity, sign or symptom that occurs after commencement of first study intervention, which is not unequivocally due to progression of disease, should be considered an AE.

All AEs must be reported on the relevant CRF and submitted to ICR-CTSU.

The severity of AEs should be graded according to the NCIC-CTC criteria v 5.0. For each AE, the highest grade observed since the last visit should be reported.

Whenever one or more toxicity/sign/symptom corresponds to a disease or a well-defined syndrome only the main disease/syndrome should be reported.

11.3. Reporting of Serious Adverse Events to ICR-CTSU

Any SAE that occurs after the first study intervention and up to 30 days following the last administration of study treatment must be reported.

All SAEs should be reported to ICR-CTSU within 24 hours of the Principal Investigator (or designated representative) becoming aware of the event, by completing the PACE-NODES SAE form and faxing to:

The ICR-CTSU safety desk
Email: **SAE-icr@icr.ac.uk**
For the attention of the PACE-NODES Trial team

As much information as possible, including the Principal Investigator's assessment of causality, must be reported to ICR-CTSU in the first instance. Additional follow up information should be reported as soon as it is available.

All SAE forms must be completed, signed and dated by the Principal Investigator or designated representative.

11.4. Review of Serious Adverse Events

The Chief Investigator (or designated representative) will assess all reported SAEs for causality and expectedness (NB. The Chief Investigator cannot down-grade the Principal Investigator's assessment of causality.)

SAEs assessed as having a causal relationship to study treatment and as being unexpected will undergo expedited reporting to the relevant authorities and all other interested parties by ICR-CTSU (see 11.5).

Sites should respond as soon as possible to requests from the Chief Investigator or designated representative (via ICR-CTSU) for further information that may be required for final assessment of an SAE.

11.5. Expedited Reporting of Related Unexpected SAEs

If an SAE is identified as being related and unexpected by the Chief Investigator it will be reported by ICR-CTSU to the main REC, the Sponsor and all other interested parties within 15 days of being notified of the event.

The Principal Investigators at all actively recruiting sites will be informed of any related unexpected SAEs occurring within the trial at appropriate intervals.

11.6. Follow up of Serious Adverse Events

SAEs should be followed up until clinical recovery is complete or until disease has stabilised. SAE outcomes should be reported to ICR-CTSU using the relevant section of the SAE form as soon as the Principal Investigator or designee becomes aware of the outcome.

11.7. Annual Safety Reporting

An annual progress report will be provided to the main REC by ICR-CTSU and copied to the Sponsor at the end of the reporting year. This will include data about related unexpected SAEs and whether any safety concerns have arisen during the reporting period.

11.8. Reporting Pregnancies

If any trial participants' partner becomes pregnant while receiving trial treatment or up to 12 months after receiving trial treatment, this should be reported to ICR-CTSU using the pregnancy reporting form. Participants who become pregnant should discontinue from trial treatment immediately. Pregnancies should be followed up until conclusion and all follow-up information should be reported to ICR-CTSU. If the outcome of the pregnancy meets the definition of serious (i.e. congenital abnormality) this should be reported to ICR-CTSU following the serious adverse event reporting procedures described above.

Figure 2: Flow diagram for SAE reporting, and action following report

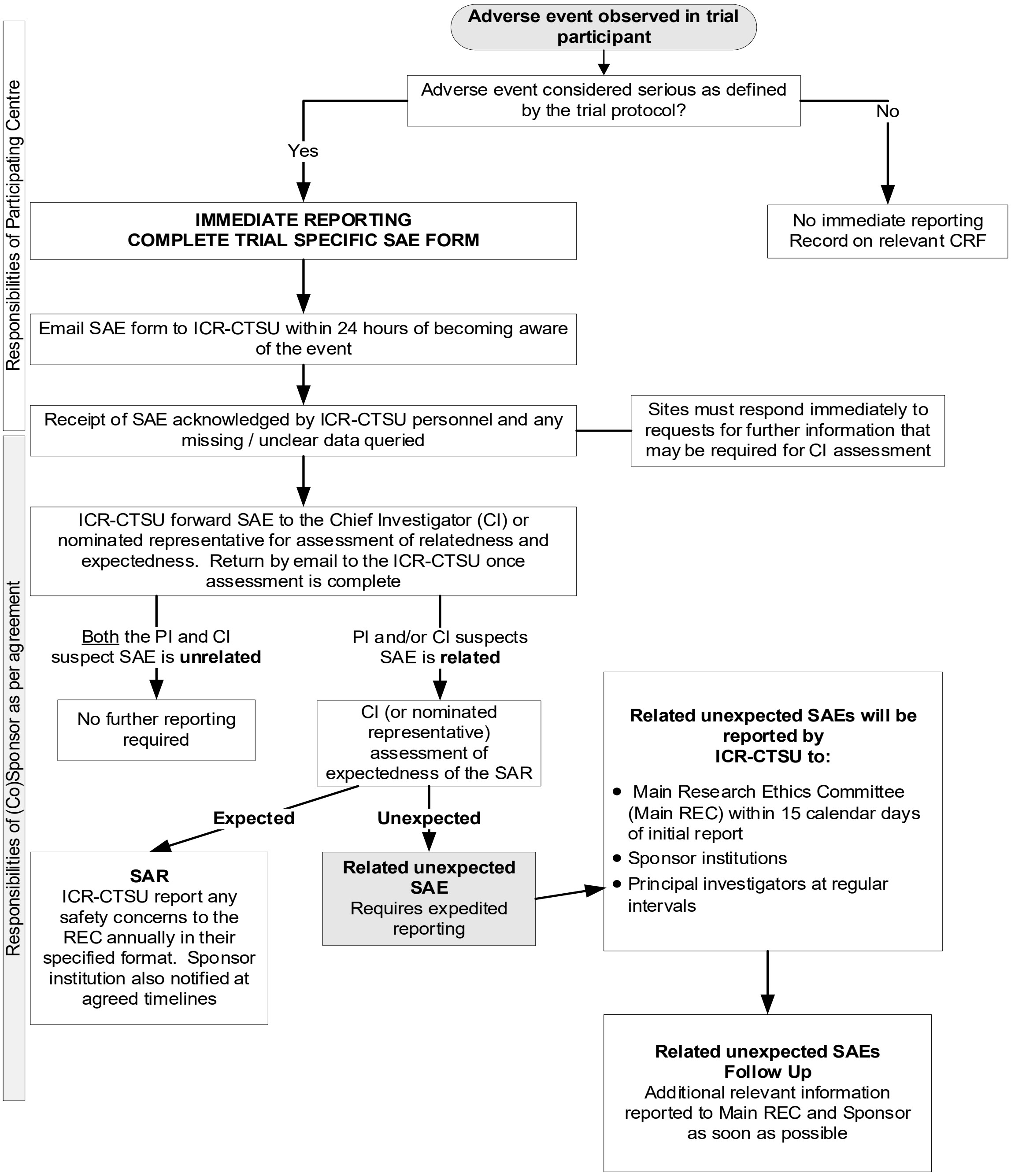

NB. All SAEs should continue to be followed up as specified above

12. STATISTICAL CONSIDERATIONS

12.1. Statistical Design and Sample Size Justification

This is a multicentre, phase III, randomised trial with the primary aim of determining superiority of PPN-SBRT over P-SBRT in terms of time to biochemical or clinical failure.

12.1.1. Sample size for primary efficacy analysis

The sample size calculation is based on assessing time to biochemical or clinical failure (primary endpoint), as defined below. In POP-RT, 5-year biochemical failure-free survival in the control arm was 81%[10]. Allowing for some improvement in outcomes over time, as suggested by recent data from PACE-B which showed very good outcomes in the lower-risk group [18] the 5-year event-free rate in the P-SBRT control arm is assumed to be 85%. The target hazard ratio (HR) for the trial is 0.6, corresponding to a 6% improvement (to 91%) in the 5-year event rate with PPN-SBRT. Although a larger effect (HR=0.23) was seen in POP-RT, the PACE-NODES design will ensure that a smaller but clinically meaningful effect is not missed. The target effect size is similar to that for PIVOTALboost (HR=0.625), which is testing the same question in the 20f setting in a lower risk group of patients, reflecting the magnitude of effect considered appropriate to counterbalance a likely increase in toxicity.

The sample size was calculated using the artsurv package (v1.1.0) within Stata software and was based on the following design parameters and assumptions:

- 1:1 allocation ratio
- staggered recruitment over 37 months in proportions of 5%, 13%, 16%, 21%, 21%, 21%, 3% per 6-month period (noting that recruitment is expected to be complete within the first month of the final period)
- 41 months minimum follow-up
- 80% power
- 5% 2-sided significance
- Expected 3% loss to follow-up at 5 years (based on CHHiP)

In order to achieve 80% power, 78 control arm events are required for the analysis (126 events expected in total), and we anticipate that this will be achieved through recruitment of 1128 patients, followed up for a minimum of approximately 3.5 years.

12.1.2. Changes to the sample size for primary efficacy analysis

The sample size was increased (from an original target of 536) in version 3.0 of the protocol. The reasons for this change were:

(i) to increase the expected event-free rate in the control arm (from 81% to 85% at 5 years) based on data from the PACE-B trial which suggested an improvement in outcomes over time; [18]

(ii) to target a slightly more conservative effect size (changing the HR from 0.5 to 0.6) to ensure that a smaller, but clinically meaningful difference could be detected. This was

particularly because use of PSMA-PET for staging within PACE-NODES may be lower than in POP-RT, since other imaging options are permissible, and this has the potential to slightly dilute any observed benefit of PPN-SBRT when compared with the earlier trial.

In recalculating the sample size, the timelines and expected rates of accrual were also adjusted based on recruitment to the trial up to that point.

12.1.3. Power for secondary toxicity analysis

This number of patients also gives adequate power for secondary toxicity comparisons, aimed at demonstrating "non-inferiority" of PPN-SBRT in terms of late gastrointestinal toxicity (CTCAE grade 2+), as follows:

Assuming a 92% evaluable rate (based on expected recurrence-free rate at 2 years and allowing for some loss to follow-up), 1038 patients would be evaluable for toxicity comparison up to 2 years. Assuming a P-SBRT toxicity rate of 13% (based on data from PACE-B [19]), the trial will have more than 85% power, with a one-sided 5% significance level, to rule out a PPN-SBRT toxicity rate of >19%, i.e. accepting an increase of up to 6% worse.

If toxicity rates are lower we will still have very good power (at least 90%) to rule out a doubling in toxicity. This relative non-inferiority margin ("a doubling") is informed by POP-RT where the cumulative late RTOG grade 2+ gastrointestinal toxicity was 6.5% for WPRT and 3.8% for PORT [20]. Unless absolute rates are very low, we will have good power (>80%) to rule out smaller relative effects (E.g. with a 10% P-SBRT toxicity rate, we would have >80% power to rule out a 50% increase, to 15%, with PPN-SBRT).

12.2. Treatment Allocation

All trial participants will receive Stereotactic Body Radiotherapy (SBRT).

Participants will be randomised between 5 fraction prostate SBRT (P-SBRT) or 5 fraction prostate and pelvic nodal SBRT (PPN-SBRT) on a 1:1 basis using minimisation with a random element. Balancing factors in the minimisation algorithm will be: centre, radiological staging method (PET-CT and/or whole body MRI; other), use of peri-rectal spacers (yes; no), use of chemotherapy/ARTA (yes; no), and planned duration of ADT (<2 years; ≥2 years).

12.3. Endpoint Definitions

12.3.1. Primary endpoint

Time to biochemical or clinical failure: defined as time from randomisation to first of biochemical failure, recommencement of androgen deprivation therapy, local recurrence, lymph node/pelvic recurrence, distant metastases or death due to prostate cancer. Recurrence identified on PSMA PET or wbMRI are included as events. For participants who are not known to have had an event at the time of analysis (including those lost to follow-up), observations will be censored on the date of most recent follow-up visit.

Biochemical failure is the most sensitive indicator of recurrent prostate cancer, encompassing clinical and radiological relapse, and is aligned with the primary outcome in PACE-C and PIVOTALboost.

Biochemical (PSA) failure will be defined following the RTOG-ASTRO Phoenix Consensus definition: an increase in serum PSA ≥2ng/ml greater than the post-treatment nadir. PSA failure will be confirmed with a second measurement (>4 weeks from the index measurement) also meeting the criteria for PSA failure.

12.3.2. Secondary endpoints

A key secondary endpoint is late gastrointestinal (GI) toxicity, grade ≥2 (CTCAE, clinician assessed).

Assessment of GI toxicity is particularly important with PPN-SBRT given the increased bowel dose compared to prostate alone radiotherapy. CTCAE is a recognised standard used in other relevant trials. Given the potential for discrepancies between clinician and patient reported toxicity [21], patient reported outcomes measures (PROMs) as a measure of the direct impact on patient quality of life [22] will provide complementary information to clinician assessed toxicity.

Other secondary endpoints include:

- Time from randomisation to first of: recommencement of androgen deprivation therapy; biochemical failure; loco-regional recurrence (recurrence in the prostate and/or pelvic lymph nodes).
- Metastatic relapse-free survival, defined as time from randomisation to distant metastases or death from prostate cancer.
- Prostate cancer-specific survival, defined as time from randomisation to death due to prostate cancer.
- Overall survival, defined as time from randomisation to death from any cause.
- Acute toxicity, occurring within the first 12 weeks of follow-up (CTCAE and RTOG), with a focus on GI and GU toxicity, grade ≥2.
- Late toxicity, reported from the 6 month visit onwards (CTCAE and RTOG), with a focus on GI and GU toxicity, grade ≥2.
- Quality of life measured using the following questionnaires: the International Prostate Symptom Score (IPSS), the 5-item version of the International Index of Erectile Function (IIEF-5), the short form Expanded Prostate Index Composite-26 (EPIC-26) and the EQ-5D-5L (see section 19.2 for further details).

- Radiotherapy planning and delivery data (PTV for prostate and pelvic nodes; adherence to dose constraints; and deviations from allocated treatment arm) to demonstrate feasibility.

- Second primary cancers occurring during trial follow-up.

12.4. Statistical Analysis Plan

All statistical analysis will be conducted by the ICR-CTSU at The Institute of Cancer Research (or in collaboration with the statistical team at ICR-CTSU). Analysis methods are outlined here, in brief. Full details will be specified in a Statistical Analysis Plan in accordance with ICR-CTSU Standard Operating Procedures.

The timing of the principal efficacy analysis is planned to be event-driven, with the required number of events expected to be achieved when all participants have a minimum of approximately 3.5 years follow-up in the trial. The Independent Data Monitoring Committee (IDMC) will monitor the accumulation of follow-up data and primary endpoint events in the trial and will advise on when the dataset is sufficiently mature for analysis.

Efficacy analyses, including the primary endpoint comparison, will include all randomised participants according to allocated treatment arm (intention-to-treat). Toxicity analyses will be performed on a per protocol analysis set, to be defined within the Statistical Analysis Plan.

The primary endpoint, as well as other time-to-event endpoints, will be presented by treatment arm using Kaplan-Meier curves; estimated 5-year event rates will be given along with 95% confidence intervals. Hazard ratios (presented with 95% confidence intervals and p-values) will be estimated from Cox proportional hazards model with adjustment for minimisation factors. Proportional hazards assumptions will be checked and, if violated, appropriate alternative methods will be applied.

Acute toxicity data will be analysed when all participants have reached the 12-week visit. Late toxicity data will be analysed when all participants have reached the 2-year visit (or been lost to follow-up/withdrawn prior to this time). Acute and late toxicity data will be summarised as grade and grade ≥2 frequencies and percentages at each time point. Worst grade adverse events occurring within each period (acute and late) will also be summarised. For each period, the proportion experiencing GI and GU toxicity (grade≥2) will be presented along with the difference in proportions with 90% confidence intervals (reflecting a one-sided 5% significance level). The relative difference between treatment groups will be estimated using logistic regression models; odds ratios with 90% confidence intervals will be presented.

Radiotherapy planning and delivery data will be presented with standard summary statistics; no formal comparisons between arms will be made. Similarly, since numbers of second primary cancers are expected to be small, these will be reported by treatment arm without formal comparison.

Any quality of life data collected from PACE-NODES participants within the SPRUCE study will be shared with PACE-NODES for analysis. Analysis of QoL data is further detailed in section 19.2.

12.5. Interim Analyses and Stopping Rules

The IDMC will review emerging safety and efficacy data at regular intervals (at least annually). Except where indicated below, data from these reviews will not normally be shared beyond the IDMC, unless the committee advise otherwise. Late toxicity will be monitored but it is unlikely that data will mature at a rate that would lead to early stopping of the trial on this basis. Likewise, no early stopping for futility (lack of efficacy) is planned.

The TMG will review radiotherapy delivery and dose constraints data, as well as any acute toxicity, of the first 15 patients randomised to PPN-SBRT. The purpose of this review will be to confirm the feasibility of the radiotherapy protocol and that treatment is being delivered within safe constraints without significant target under coverage. This will allow early modification of the protocol should any areas of concern be highlighted by this data. Recruitment will not be paused whilst this analysis is taking place.

To protect against unexpectedly high toxicity, the IDMC will review acute toxicity data once 82 patients in the PPN-SBRT arm have been treated and followed up for 12 weeks in the trial (anticipated timing of analysis 15-18 months from recruitment initiation). The primary purpose of the review will be to assess whether acute GI and GU toxicity in the PPN-SBRT arm are within acceptable limits. A cohort of 82 reflects a balance between ensuring there is sufficient data for the committee to make a reasonable assessment of acute toxicity, whilst affording the opportunity for the IDMC to recommend stopping trial recruitment at a relatively early stage, should they have any major concerns. The proportion of patients experiencing CTCAE grade 2+ GI and GU symptoms by 12 week follow-up will be considered in relation to safety data emerging from other similar trials, as well as data from the P-SBRT arm. IDMC recommendations regarding continuation and/or modification of the trial will be based on the following guidelines, which will be discussed and agreed with the committee ahead of the analysis:

i. Acute CTCAE grade 2+ GU symptoms were reported in 31% of patients in the PACE-B SBRT arm by 12 weeks [1]; if grade 2+ GU symptoms are reported in 37 or more (≥45%) of the 82 PPN-SBRT patients, this would be cause for concern. A 90% confidence interval would not be able to exclude a rate of 55%.
ii. Acute CTCAE grade 2+ GI symptoms were reported in 16% of patients in the PACE-B SBRT arm by 12 weeks [1] ; if grade 2+ GI symptoms are reported in 25 or more (≥30%) of the 82 PPN-SBRT patients, this would be cause for concern. A 90% confidence interval would not be able to exclude a rate of 40%.

Unless the IDMC advise otherwise, recruitment will not be paused whilst this interim toxicity analysis is taking place.

The final analysis of acute toxicity data will be performed when all participants have reached the 12-week visit and, with the permission of the IDMC, results will be released for

presentation/publication to help inform planning of future studies. Similarly, an analysis of late toxicity data will be performed when all participants have reached the 2-year visit and may be published ahead of the primary efficacy analysis, subject to permission from the IDMC.

13. TRIAL MANAGEMENT

13.1. Trial Management Group (TMG)

A Trial Management Group (TMG) will be set up and will include the Chief Investigator, ICR-CTSU Scientific Lead, clinical leads, co-investigators and identified collaborators, the Trial Statistician and Clinical Trial Manager. Principal Investigators and key study personnel will be invited to join the TMG as appropriate to ensure representation from a range of sites and professional groups. Where possible, membership will include a lay/consumer representative. The TMG will meet at regular intervals, and at least annually. Notwithstanding the legal obligations of the Sponsor and Chief Investigator, the TMG have operational responsibility for the conduct of the trial. The Committee's terms of reference, roles and responsibilities will be defined in a charter issued by ICR-CTSU.

13.2. Trial Steering Committee (TSC)

A Trial Steering Committee (TSC) will be set up and will comprise an independent Chairman and at least two further independent members with clinical or statistical expertise (at least one member must be a statistician). The TSC will meet at regular intervals, and at least annually. The TSC will provide expert independent oversight of the trial on behalf of the Sponsor and funder. The Committee's terms of reference, roles and responsibilities will be defined in charter issued by ICR-CTSU.

13.3. Independent Data Monitoring Committee (IDMC)

An Independent Data Monitoring Committee (IDMC) will be set up to monitor the progress of the trial and will comprise a Chairman and at least two further members with clinical or statistical expertise (at least one member must be a statistician). Membership of the IDMC will be proposed by the TMG and approved by the TSC.

The IDMC will meet in confidence at regular intervals, and at least annually. A summary of findings and any recommendations will be produced following each meeting. This summary will be submitted to the TMG and TSC, and if required, the main REC and the MHRA.

The IDMC will reserve the right to release any data on outcomes or side-effects through the TSC to the TMG (and if appropriate to participants) if it determines at any stage that the combined evidence from this and other studies justifies it.

The Committee's terms of reference, roles and responsibilities will be defined in a charter issued by ICR-CTSU.

14. RESEARCH GOVERNANCE

14.1. Sponsor Responsibilities

The Sponsor of this clinical trial is the Institute of Cancer Research (ICR).
As and when applicable, a coordinating group in each participating (non-UK) country will be delegated responsibility for trial initiation and conduct in that country on behalf of the Sponsor, as defined in an agreement between the Sponsor and the coordinating group.

14.2. Participating Site Responsibilities

Responsibilities of participating sites are defined in an agreement between the individual participating site and the Sponsor (UK) or, as and when applicable, the coordinating group delegated that responsibility by the Sponsor (non-UK).

15. TRIAL ADMINISTRATION & LOGISTICS

15.1. Site Activation

Before activating the trial, participating sites are required to sign an agreement accepting responsibility for all trial activity which takes place within their site.

Sites may commence recruitment once the site agreement has been signed by all required signatories, the required trial documentation is in place (as specified by ICR-CTSU) and a site initiation (visit or teleconference) has taken place. Site initiation visits will be conducted at sites where the Principal Investigator has requested one or where ICR-CTSU deems it is appropriate.

15.2. Data Acquisition

Electronic (e) Case Report Forms (CRF) will be used for the collection of trial data. ICR-CTSU will provide guidance to sites to aid the completion of the eCRFs. The Trial Management Group reserves the right to amend or add to the eCRF template as appropriate. Such changes do not constitute a protocol amendment, and revised or additional forms should be used by sites in accordance with the guidelines provided by ICR-CTSU.

15.3. Central Data Monitoring

Once data has been entered on the eCRF by the site personnel, ICR-CTSU will review it for compliance with the protocol, and for inconsistent or missing data. Should any missing data or data anomalies be found, queries will be raised for resolution by the site.

Any systematic inconsistencies identified through central data monitoring may trigger an on-site monitoring visit.

15.4. On-Site Monitoring

If a monitoring visit is required, ICR-CTSU will contact the site to arrange the visit. Once a date has been confirmed, the site should ensure that full patient notes, including electronic notes, of participants selected for source data verification are available for monitoring.

ICR-CTSU staff conducting on-site monitoring will review essential documentation and carry out source data verification to confirm compliance with the protocol. If any problems are detected during the course of the monitoring visit, ICR-CTSU will work with the Principal Investigator or delegated individual to resolve issues and determine appropriate action.

15.5. Completion of the Study and Definition of Study End Date

The study end date is deemed to be the date of last data capture.

15.6. Archiving

Essential trial documents should be retained according to local policy and for a sufficient period for possible inspection by the regulatory authorities (at least 5 years after the date of last data capture). Documents should be securely stored and access restricted to authorised personnel.

16. PATIENT PROTECTION AND ETHICAL CONSIDERATIONS

16.1. Risk Assessment and Approval

This trial has been formally assessed for risk and approved by the Sponsor's Committee for Clinical Research.

16.2. Public and Patient Involvement

Patient advocate members were involved in protocol design including methodology, sample collection, patient information and consent forms and are represented on the TMG.

16.3. Ethics Approvals

The trial will not commence at any participating site until the required approvals are in place. In the UK, ICR-CTSU, on behalf of the Sponsor, will ensure that the trial has received ethics approval from a research ethics committee (REC) for multi-centre trials, HRA approval and relevant NHS Permissions. Before recruiting patients, the Principal Investigator at each site is responsible for obtaining local approvals.

As and when applicable, the coordinating group in each country, on behalf of the Sponsor, will ensure that the trial has received all relevant ethical, regulatory and institutional approval prior to the recruitment of any patients.

16.4. Trial Conduct

This trial will be conducted according to the approved protocol and its amendments, supplementary guidance and manuals supplied by the Sponsor and in accordance with the UK Policy Framework for Health and Social Care and the principles of GCP.

16.5. Informed Consent

The Principal Investigator retains overall responsibility for the conduct of research at their site; this includes the taking of informed consent of participants. They must ensure that any clinician delegated responsibility to participate in the informed consent process is duly authorised, trained and competent to do so in accordance with the ethically approved protocol, principles of Good Clinical Practice and Declaration of Helsinki.

Patients should be asked to sign the current ethics approved PACE-NODES consent form at trial entry after receiving both verbal and written information about the trial, having been given sufficient time to consider this information. All consent forms must be countersigned by the Principal Investigator or a designated clinician. A signature log of delegated responsibilities, listing the designated clinicians and the circumstances under which they may countersign consent forms, must be maintained at the participating site. This log, together with original copies of all signed patient consent forms, should be retained in the Site Investigator File and must be available for inspection. The current ethics approved PACE-NODES patient information sheets should be provided in addition to any standard patient information sheets that are provided by the site and used in routine practice.

16.6. Patient Confidentiality

UK patients will be asked to consent to their full name being collected at trial entry in addition to their date of birth, hospital number, postcode and NHS number or equivalent to allow linkage with routinely collected NHS data and ensure accuracy in handling biological samples. For international sites only initials and partial date of birth will be collected.

Each investigator should keep a separate log of all participants' Trial IDs, names, addresses and hospital numbers. The investigator must retain trial documents (e.g. participants' written consent forms) in strict confidence. The investigator must ensure the participants' confidentiality is maintained at all times.

Representatives of ICR-CTSU will require access to participants' hospital notes for quality assurance purposes. ICR-CTSU will maintain the confidentiality of participants at all times and will not reproduce or disclose any information by which participants could be identified.

16.7. Data Protection

All investigators and trials staff must comply with applicable data protection laws at all times.

16.8. *Liability*

Indemnity to meet the potential legal liability of investigators participating in this trial is provided by the usual NHS indemnity arrangements.

As and when applicable, the coordinating group in each country will ensure that appropriate indemnity arrangements are place to meet the potential legal liabilities of investigators conducting the trial.

17. FINANCIAL MATTERS

This trial is investigator designed and led. ICR has received funding from Prostate Cancer UK for the central coordination of the trial. The trial meets the criteria for R&D support as outlined in the Statement of Partnership on Non-Commercial R&D in the NHS in England. The trial is part of the National Institute for Health Research Clinical Research Network (NIHR CRN) portfolio by virtue of its funding by an NIHR non-commercial partner. NIHR CRN resources should therefore be made available for the trial to cover UK specific research costs

18. PUBLICATION POLICY

The main trial results will be published in a peer-reviewed journal, on behalf of all collaborators. The manuscript will be prepared by a writing group, consisting of members of the TMG. Participating clinicians may be selected to join the writing group on the basis of intellectual and time input. All participating clinicians will be acknowledged in the publication.

Any presentations and publications relating to the trial must be authorised by the TMG. Authorship of any secondary publications e.g. those relating to sub-studies, will reflect intellectual and time input into these studies. Authorship of all publication will usually be in accordance with ICMJE guidance.

No investigator may present or attempt to publish data relating to the PACE-NODES trial without prior permission from the TMG.

It is an expectation that all publications relating to the trial are published as “open-access”.

19. ASSOCIATED STUDIES

19.1. *Translational Study*

All participants will be asked for consent to collect diagnostic prostate biopsy samples (FFPE blocks) for future research. These will be collected at the end of recruitment and batch shipped (ambient temperature, Royal mail) to the Translational Radiobiology Lab, University of Manchester for storage.

19.2. Quality of Life Study

All participants will be asked to provide consent for the quality of life study, but this will be optional.

Quality of life (QL) will be evaluated using the following questionnaires: International prostate symptom score (IPSS), International Index of Erectile Function (IIEF-5), the Expanded Prostate Index Composite-26 (EPIC-26) short form questionnaire, and the EQ-5D-5L.

The IPSS questionnaire is a validated diagnostic tool (7 items) and will be used to assess urinary and bowel incontinence [23].

The IIEF-5 is an abridged 5-item version of the 15-item International Index of Erectile Function (IIEF) and is a validated diagnostic tool for erectile dysfunction [24].

The EPIC questionnaire (in its version with 26 items) was selected as it best represents typical symptoms after radiotherapy in prostate cancer patients [25]. The EPIC domains for urinary incontinence, urinary irritative/obstructive, bowel and sexual have been advocated within the standard set of patient reported outcomes for men with localised prostate cancer by ICHOM65 [26].

The EQ-5D is one of the most commonly used generic questionnaires to measure health-related QoL. The EQ-5D questionnaire consists of a questionnaire and a visual analogue scale (EQ-VAS). Five dimensions are assessed (mobility, self-care, usual activities, pain/discomfort, anxiety/depression) and the 5L version of the questionnaire, in which patients score each dimension on a 5-level severity scale, will be used. This has been shown to have similar or better measurement properties than the EQ-5D-3L in a systematic review of comparative studies [27]. The EQ-VAS is a self-rated health status using a VAS. The EQ-VAS records the subject's perceptions of their own current overall health and can be used to monitor changes with time.

QoL questionnaires will be completed at baseline, and then at 4 weeks, 6, 12, 24 and 60 month follow-up visit, post-radiotherapy completion. Use of a web-based data capture system, whereby participants can directly enter responses online, is planned.

A QoL analysis plan will be developed in consultation with the TMG with key endpoints for each questionnaire. Standard algorithms will be used to derive scores and handle missing data. Changes from baseline at each time point will be compared within groups as well as between treatment groups (by means of ordinal logistic regressions or ANCOVA models). Analyses to account for the longitudinal nature of the data may be used.

Participation in the ICR-CTSU study within a trial investigating electronic collection of patient reported outcomes, SPRUCE, is permitted.

20. REFERENCES

1. Brand, D.H., et al., *Intensity-modulated fractionated radiotherapy versus stereotactic body radiotherapy for prostate cancer (PACE-B): acute toxicity findings from an international, randomised, open-label, phase 3, non-inferiority trial.* The Lancet Oncology, 2019. **20**(11): p. 1531-1543.
2. Catton, C.N., et al., *Randomized Trial of a Hypofractionated Radiation Regimen for the Treatment of Localized Prostate Cancer.* Journal of Clinical Oncology, 2017. **35**(17): p. 1884-1890.
3. Dearnaley, D., et al., *Conventional versus hypofractionated high-dose intensity-modulated radiotherapy for prostate cancer: 5-year outcomes of the randomised, non-inferiority, phase 3 CHHiP trial.* The Lancet Oncology, 2016. **17**(8): p. 1047-1060.
4. Amini, A., et al., *Survival Outcomes of Whole-Pelvic Versus Prostate-Only Radiation Therapy for High-Risk Prostate Cancer Patients With Use of the National Cancer Data Base.* International Journal of Radiation Oncology*Biology*Physics, 2015. **93**(5): p. 1052-1063.
5. Dearnaley, D., et al., *Toxicity and Patient-Reported Outcomes of a Phase 2 Randomized Trial of Prostate and Pelvic Lymph Node Versus Prostate only Radiotherapy in Advanced Localised Prostate Cancer (PIVOTAL).* International Journal of Radiation Oncology*Biology*Physics, 2019. **103**(3): p. 605-617.
6. Lawton, C.A., et al., *An Update of the Phase III Trial Comparing Whole Pelvic to Prostate Only Radiotherapy and Neoadjuvant to Adjuvant Total Androgen Suppression: Updated Analysis of RTOG 94-13, With Emphasis on Unexpected Hormone/Radiation Interactions.* International Journal of Radiation Oncology*Biology*Physics, 2007. **69**(3): p. 646-655.
7. Reis Ferreira, M., et al., *Phase 1/2 Dose-Escalation Study of the Use of Intensity Modulated Radiation Therapy to Treat the Prostate and Pelvic Nodes in Patients With Prostate Cancer.* International Journal of Radiation Oncology*Biology*Physics, 2017. **99**(5): p. 1234-1242.
8. Widmark, A., et al., *Ultra-hypofractionated versus conventionally fractionated radiotherapy for prostate cancer: 5-year outcomes of the HYPO-RT-PC randomised, non-inferiority, phase 3 trial.* The Lancet, 2019. **394**(10196): p. 385-395.
9. Kothari, G., et al., *Stereotactic Body Radiotherapy for Primary Prostate Cancer.* Technol Cancer Res Treat, 2018. **17**: p. 1533033818789633.
10. Murthy, V., et al., *Prostate-Only Versus Whole-Pelvic Radiation Therapy in High-Risk and Very High-Risk Prostate Cancer (POP-RT): Outcomes From Phase III Randomized Controlled Trial.* Journal of Clinical Oncology, 2021. **39**(11): p. 1234-1242.
11. Roach, M., 3rd, et al., *Predicting the risk of lymph node involvement using the pre-treatment prostate specific antigen and Gleason score in men with clinically localized prostate cancer.* Int J Radiat Oncol Biol Phys, 1994. **28**(1): p. 33-7.
12. Bauman, G., et al., *A Phase 1/2 Trial of Brief Androgen Suppression and Stereotactic Radiation Therapy (FASTR) for High-Risk Prostate Cancer.* International Journal of Radiation Oncology*Biology*Physics, 2015. **92**(4): p. 856-862.
13. Callan, L., et al., *A Phase I/II Trial of Fairly Brief Androgen Suppression and Stereotactic Radiation Therapy for High-Risk Prostate Cancer (FASTR-2): Preliminary Results and Toxicity Analysis.* Adv Radiat Oncol, 2019. **4**(4): p. 668-673.
14. Musunuru, H.B., et al., *Phase 1-2 Study of Stereotactic Ablative Radiotherapy Including Regional Lymph Node Irradiation in Patients With High-Risk Prostate Cancer (SATURN): Early Toxicity and Quality of Life.* International Journal of Radiation Oncology*Biology*Physics, 2018. **102**(5): p. 1438-1447.

15. Murthy, V., et al., *Study protocol of a randomised controlled trial of prostate radiotherapy in high-risk and node-positive disease comparing moderate and extreme hypofractionation (PRIME TRIAL).* BMJ Open, 2020. **10**(2): p. e034623.
16. Fairmichael, C., et al., *The stereotactic prostate radiotherapy (SPORT) trial: A randomized feasibility study comparing prostate SABR to prostate and pelvic nodal SABR.* Journal of Clinical Oncology, 2021. **39**(6_suppl): p. 248-248.
17. Roach, M., 3rd, et al., *Defining biochemical failure following radiotherapy with or without hormonal therapy in men with clinically localized prostate cancer: recommendations of the RTOG-ASTRO Phoenix Consensus Conference.* Int J Radiat Oncol Biol Phys, 2006. **65**(4): p. 965-74.
18. van As, N., et al., *5-Year Outcomes from PACE B: An International Phase III Randomized Controlled Trial Comparing Stereotactic Body Radiotherapy (SBRT) vs. Conventionally Fractionated or Moderately Hypo Fractionated External Beam Radiotherapy for Localized Prostate Cancer.* International Journal of Radiation Oncology*Biology*Physics, 2023. **117**(4): p. e2-e3.
19. Tree, A.C., et al., *Intensity-modulated radiotherapy versus stereotactic body radiotherapy for prostate cancer (PACE-B): 2-year toxicity results from an open-label, randomised, phase 3, non-inferiority trial.* Lancet Oncol, 2022. **23**(10): p. 1308-1320.
20. Murthy, V., et al., *Late toxicity and quality of life with prostate only or whole pelvic radiation therapy in high risk prostate cancer (POP-RT): A randomised trial.* Radiotherapy and Oncology, 2020. **145**: p. 71-80.
21. Basch, E., et al., *Adverse Symptom Event Reporting by Patients vs Clinicians: Relationships With Clinical Outcomes.* JNCI: Journal of the National Cancer Institute, 2009. **101**(23): p. 1624-1632.
22. Snyder, C.F., et al., *Implementing patient-reported outcomes assessment in clinical practice: a review of the options and considerations.* Quality of Life Research, 2012. **21**(8): p. 1305-1314.
23. Barry, M.J., et al., *The American Urological Association symptom index for benign prostatic hyperplasia. The Measurement Committee of the American Urological Association.* J Urol, 1992. **148**(5): p. 1549-57; discussion 1564.
24. Rosen, R.C., et al., *Development and evaluation of an abridged, 5-item version of the International Index of Erectile Function (IIEF-5) as a diagnostic tool for erectile dysfunction.* Int J Impot Res, 1999. **11**(6): p. 319-26.
25. Wei, J.T., et al., *Development and validation of the expanded prostate cancer index composite (EPIC) for comprehensive assessment of health-related quality of life in men with prostate cancer.* Urology, 2000. **56**(6): p. 899-905.
26. Martin, N.E., et al., *Defining a standard set of patient-centered outcomes for men with localized prostate cancer.* Eur Urol, 2015. **67**(3): p. 460-7.
27. Buchholz, I., et al., *A Systematic Review of Studies Comparing the Measurement Properties of the Three-Level and Five-Level Versions of the EQ-5D.* Pharmacoeconomics, 2018. **36**(6): p. 645-661.

A1. WHO performance status

Grade	Performance Status
0	Able to carry out all normal activity without restriction.
1	Restricted in physically strenuous activity but ambulatory and able to carry out light work.
2	Ambulatory and capable of all self-care but unable to carry out any work; up and about more than 50% of waking hours.
3	Capable of only limited self-care; confined to bed or chair more than 50% of waking hours.
4	Completely disabled; cannot carry on any self-care; totally confined to bed or chair.

A2. RTOG Toxicity Scales

Instructions:

1. Toxicity grade should reflect the most severe degree occurring during the evaluated period, not an average.
2. When two criteria are available for similar toxicities, the one resulting in the more severe grade should be used.
3. Toxicity grade = 5 if that toxicity caused death of the patient.
4. An accurate baseline prior to start of therapy is necessary.

Definitions:

Diarrhoea is defined as a clinical syndrome characterised by frequent loose bowel movements without associated rectal irritation (tenesmus)

Proctitis is defined as a clinical syndrome characterised by rectal irritation or urgency (tenesmus), presence of mucous or blood in the stool and, in some patients, with frequent, sometimes loose bowel movements.

Cystitis is defined as a syndrome characterised by irritative bladder symptoms such as frequency, dysuria and nocturia. Haematuria may or may not be a part of the clinical picture of cystitis.

Acute Toxicity [To be used from baseline to 12 week follow up visit]:

Bladder changes – cystitis/frequency:

N.B. Patients on medication for symptoms at baseline will be scored at grade 2 at that time point and then for on treatment assessments scored as grade 1 as long as medication remains the same. Should they require an increase in number or dose of bladder medication they will be escalated to grade 2 (or above if required)

Grade 0: No symptoms

Grade 1: Frequency of urination or nocturia twice pre-treatment habit/dysuria, urgency not requiring medication.

Grade 2: Frequency of urination or nocturia which is less frequent than every hour. Dysuria, urgency, bladder spasm requiring local anaesthetic.

Grade 3: Frequency with urgency and nocturia hourly or more frequently/dysuria, pelvic pain or bladder spasm requiring regular, frequent narcotic/gross haematuria with/without clot passage.

Grade 4: Haematuria requiring transfusion/acute bladder obstruction not secondary to clot passage, ulceration or necrosis.

Grade 5: Death directly due to radiation morbidity.

Bowel changes

Grade 0: No symptoms

Grade 1: Increased frequency or change in quality of bowel habits not requiring medication/rectal discomfort not requiring analgesics.

Grade 2: Diarrhoea requiring parasympatholytic drugs/mucous discharge not necessitating sanitary pads/rectal abdominal pain requiring analgesics.

Grade 3: Diarrhoea requiring parenteral support/severe mucous or blood discharge necessitating sanitary pads/abdominal distention (flat plate radiograph demonstrates distended bowel loops).

Grade 4: Acute or subacute obstruction, fistula or perforation/GI bleeding requiring transfusion/abdominal pain or tenesmus requiring tube decompression or bowel diversion.

Grade 5: Death directly due to radiation morbidity.

Late Toxicity [To be used from 6 month follow up visit onwards]:

Grade 0: No symptoms

Grade 1: Minor symptoms requiring no treatment

Grade 2: Symptoms responding to a simple outpatient management, lifestyle (performance status not affected)

Grade 3: Distressing symptoms altering patient's lifestyle (performance status). Hospitalisation for diagnosis or minor surgical intervention (such as urethral dilatation) may be required.

Grade 4: Major surgical intervention (such as laparotomy, colostomy, and cystectomy) or prolonged hospitalisation required.

Grade 5: Fatal complications

A3. Expected serious adverse events

The following are possible anticipated treatment related AEs (i.e. expected occurrences), ≤CTCAE Grade 3, which are not subject to expedited reporting to ICR-CTSU but all such events should be reported in the appropriate section of the CRF.

SBRT

- Urinary toxicities:
  - Urinary frequency/urgency/nocturia
  - Urinary retention
  - Urinary obstruction/strictures
  - Haematuria
  - Cystitis/bladder spasms
  - Urinary incontinence/leakage
  - Pain (prostate, urinary/dysuria)
- GI Toxicities:
  - Pain (rectal, pelvic, abdominal)
  - Diarrhoea
  - Constipation
  - Rectal bleeding/ulcer
  - Fistula
  - Proctitis
  - Bowel obstruction or perforation
- Sexual function
  - Erectile dysfunction
  - Decreased volume of ejaculate/absence of ejaculate
  - Decreased libido
- Dermatology/Skin
  - Rash
  - Hair loss in treatment area
- Bone fractures
- Lymphodema
- Related to fiducial marker insertion
  - Bleeding
  - Sepsis (urinary and systemic)
  - Pain

Additional expected events related to use of ADT as standard of care

- Hot flushes
- Fatigue
- Mood changes
- Weight gain
- Erectile dysfunction
- Loss of libido
- Reduction in bone mineral density
- Cardiovascular events (MI, CVA)

A4. GLOSSARY

AE	Adverse Event
ARTA	Androgen Receptor Target Agents
CI	Chief Investigator
CRF	Case Report Form
DCF	Data Capture Form
HR	Hazard Ratio
ICR	The Institute Of Cancer Research
IDMC	Independent Data Monitoring Committee
MDT	Multi-disciplinary team
NCI CTCAE	National Cancer Institute Common Terminology Criteria for Adverse Events
PI	Principal Investigator
PIS	Patient Information Sheet
QoL	Quality of Life
R&D	Research and Development
RCT	Randomised controlled trial
SAE	Serious Adverse Event
SAR	Serious Adverse Reaction
SPRUCE	**S**tudy within a trial of electronic versus paper based **P**atient **R**eported o**U**tcomes **C**oll**E**ction (REC Ref: 21/WM/0223)
TMG	Trial Management Group
TSC	Trial Steering Committee
WHO	World Health Organisation

This protocol is a controlled document and should not be copied, distributed or reproduced without the written permission of the ICR-CTSU
