# Supplementary material for: PACE-NODES: A phase III randomised trial of 5 fraction prostate stereotactic body radiotherapy (SBRT) versus 5 fraction prostate and pelvic nodal SBRT
Source: Clin Transl Radiat Oncol. 2026 Feb 11;58:101126. doi: 10.1016/j.ctro.2026.101126 (PMC12925429; doi:10.1016/j.ctro.2026.101126)
Supplement: Supplementary Data 2 [file mmc2.pdf]

A phase III randomised trial of 5 fraction prostate SBRT versus 5 fraction prostate and pelvic nodal SBRT

RADIOTHERAPY PLANNING AND DELIVERY GUIDELINES

Version: 1.2

Dated: 31/01/2023

Chief Investigator:	Dr Nicholas van As
Clinical Co-ordinators:	Dr Angela Pathmanathan, Prof Suneil Jain
Sponsor:	The Institute of Cancer Research
Funders:	Prostate Cancer UK (PCUK)
Coordinating Trials Unit:	ICR Clinical Trials and Statistics Unit (ICR-CTSU)
	The Institute of Cancer Research
Main REC Reference Number:	22/LO/0263
ISRCTN:	NCT05613023

This is a controlled document which should be referred to in conjunction with the PACE-NODES protocol and should not be copied, distributed or reproduced without the written permission of the PACE-NODES Trials Office – PACE-NODES-icrctsu@icr.ac.uk

ADMINISTRATION

Clinical Coordination

Prof Nicholas van As
(Chief Investigator)
Royal Marsden Hospital
Fulham Road, London, SW3 6JJ
Nicholas.VanAs@rmh.nhs.uk

Dr Angela Pathmanathan
(Co-Clinical Lead)
Royal Marsden Hospital
Sutton, SM2 5PT
Angela.Pathmanathan@icr.ac.uk

Prof Suneil Jain
(Co-Clinical Lead)
Northern Ireland Cancer Centre
Belfast City Hospital
Lisburn Road, Belfast, BT9 7AB
s.jain@qub.ac.uk

ICR-CTSU Methodology Lead:
Prof Emma Hall
Tel: 020 8722 4013
Emma.Hall@icr.ac.uk

ICR-CTSU Statisticians:
Fay Cafferty / Monisha Dewan
Tel: 020 3437 6794 / 020 8722 4008
PACE-NODES-icrctsu@icr.ac.uk

ICR-CTSU Clinical Trial Programme Manager:
Stephanie Burnett
Tel: 020 8722 4261
PACE-NODES-icrctsu@icr.ac.uk

PACE-NODES Senior Trial Manager:
Stephanie Brown
Tel: 020 8722 4467
PACE-NODES-icrctsu@icr.ac.uk

Any questions relating to the Radiotherapy Planning document should be addressed in the first instance to the PACE-NODES RTQA team:

Radiotherapy Quality Assurance Team RTQA Physicists, email: pace-nodes.rtqa@nhs.net

Olivia Naismith
Royal Marsden Hospital
Fulham Road, London, SW3 6JJ

Table of Contents

1. Introduction and Trial Summary 5
1.1 Trial Schema 7
2 Pre-planning Procedures 8
2.1 Fiducial Markers 8
2.2 Hydrogel Spacers 8
2.3 Planning CT and MRI scans 8
2.4 Patient Preparation and Positioning 8
3 Organs at Risk and target volume definitions 9
3.1 Target Volume Definition 9
3.2 Organs at Risk 11
3.3 Structure Naming Convention 12
4 Margins for Planning Target Volumes 13
4.1 PTV Margins 13
5 External Beam Radiotherapy Planning Guidelines 13
5.1 Radiotherapy Technique 13
5.2 Prescribed Dose and Fractionation 13
5.3 Definition of PTVs for Dose Reporting with their Dose Constraints 14
5.4 Normal Tissue Dose Constraints for Organs at Risk 15
5.5 Dose-Volume Variations 15
6 Radiotherapy Delivery 16
6.1 Treatment Scheduling 16
6.2 Treatment Verification 16
7 Documentation on Completion of Radiotherapy 18
8 Radiotherapy Quality Assurance 18
8.1 Radiotherapy Quality Assurance Overview 18
8.2 Pre-trial QA 19
8.3 On-Trial QA 21
8.4 Ongoing Data Collection 22
8.5 DICOM Data Export 22
9 References 24

Major Revisions since previous Version

Version No.	Major Revisions
1.1	Section 5.3.2 report all nodal PTV dose objectives to a cropped structure, out_PTVn
1.2	Section 6.1 added instructions for missed treatment fractions<br>Section 6.2 amended IGRT matching time and re-imaging requirements<br>Section 6.2 amended actions for prostate/nodes mismatch

1. Introduction and Trial Summary

This document supplements the PACE-NODES trial protocol, and describes the radiotherapy procedures and radiotherapy quality assurance (QA) programme for the PACE-NODES trial.

All patients will receive stereotactic body radiotherapy (SBRT) to the prostate +/- pelvic lymph nodes. Both are delivered with daily online image-guided radiotherapy (IGRT). Various delivery techniques are allowed (e.g. VMAT, helical tomotherapy, robotic gantry, MR-linac), and all will be referred to as SBRT in this document unless specifically detailed.

PROTOCOL TITLE	**PACE-NODES:** A phase III randomised trial of 5 fraction prostate SBRT (P-SBRT) versus 5 fraction prostate and pelvic nodal SBRT (PPN-SBRT)
TARGET DISEASE	• Histopathological confirmation of prostate adenocarcinoma with Gleason/ISUP grade group scoring<br>• Patients planned for 12-36 months androgen deprivation therapy<br>• High risk localised prostate cancer as defined by:<br>- Gleason 8-10 (grade groups 4 and 5) and/or<br>- Stage T3a/b or T4 and/or<br>- PSA > 20 ng/ml<br>• Multi-parametric MRI of the pelvis- to include at least one functional MRI sequence in addition to T2W imaging within twelve months of randomisation<br>• Radiological staging to exclude metastatic disease prior to starting ADT, with one of the following- PSMA PET-CT, fluciclovine/choline PET-CT, whole-body MRI, bone scan, CT of chest, abdomen and pelvis (imaging method as per local practice/standard of care)
TRIAL OBJECTIVES	The primary objective is to determine whether PPN-SBRT has superior biochemical/clinical-failure free rate (reduces the risk of biochemical or clinical failure by 50% or more) than P-SBRT, in patients with high risk localised prostate cancer
TRIAL DESIGN	PACE-NODES is a multicentre phase III randomised controlled trial
TRIAL POPULATION	Patients with high risk localised prostate cancer, deemed suitable for SBRT radiotherapy and planned for 12-36 months androgen deprivation therapy

RECRUITMENT TARGET	The aim is to recruit 536 participants; 268 into each arm of the study
TRIAL TREATMENT	Patients will be allocated to one of two treatment arms:<br>• Prostate alone SBRT (P-SBRT) to receive 36.25 Gy in 5 fractions to the prostate and seminal vesicles<br>• Prostate and pelvic node SBRT (PPN-SBRT) to receive 36.25 Gy in 5 fractions to the prostate and seminal vesicles and 25 Gy in 5 fractions to pelvic nodes
PRIMARY ENDPOINT	Time to biochemical or clinical failure as defined by time from randomisation to the first biochemical failure, local recurrence, lymph node/pelvic recurrence, distant metastases, recommencement of androgen deprivation therapy or death due to prostate cancer
SECONDARY ENDPOINTS	1. Clinical reported acute and late toxicity using CTCAE version 5.0 and RTOG criteria. Focus will be given to GU and GI Grade 2 or higher (G2+) toxicities.<br>2. Metastatic relapse-free survival, prostate cancer-specific survival and overall survival<br>3. PROMs as assessed by IPSS, EPIC-26, EQ-5D and IIEF-5<br>4. Adherence to radiotherapy protocol
FOLLOW UP	Evaluation during and after treatment will be as follows:<br>1. Acute toxicity will be assessed at the final fraction, then 2, 4, 8 and 12 weeks after end of treatment<br>2. Late toxicity will be assessed at 6, 12, 18, 24, 36, 48 and 60 months<br>3. Quality of life questionnaires will be completed at 4 weeks after treatment and then at 6, 12, 24 and 60 months after the end of treatment<br>4. PSA (for determination of biochemical failure) will be collected at 6 months following completion of SBRT, then 6-monthly for 5 years<br>Long-term data capture will be pursued through routine data sources.

1.1 Trial Schema

PACE-NODES is a multi-centre, randomised phase III trial which recruits men with high risk localised prostate cancer to receive either 5 fraction prostate SBRT (P-SBRT) or prostate and pelvic node SBRT (PPN-SBRT).

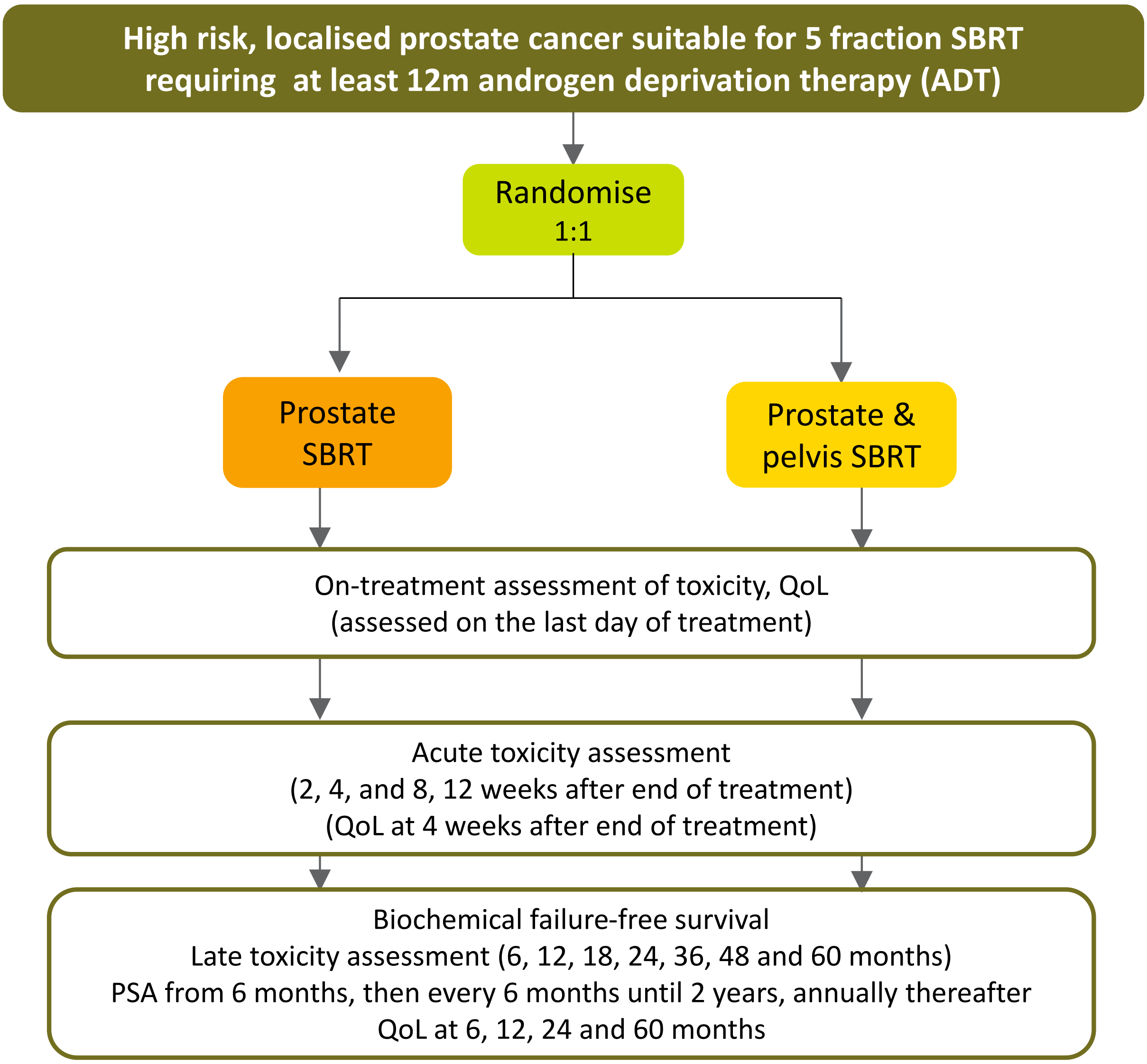

- Toxicity will be assessed using Radiation Therapy Oncology Group (RTOG) grading and the National Cancer Institute Common Terminology Criteria for Adverse Events (CTCAE) version 5.0.
- Patient reported International Prostate Symptom Score (IPSS), Expanded Prostate Index Composite-26 (EPIC-26) and EuroQol-5D (EQ-5D) QOL will be measured at baseline, 4 weeks after treatment and then at 6, 12, 24 and 60 months from the end of radiotherapy. The International Index of Erectile Function 5 (IIEF-5) will be measured at 60 months.
- All participants will be followed up for at least 3.5 years with trial specific follow-up to 5 years.

2 Pre-planning Procedures

2.1 Fiducial Markers

It is strongly recommended that all patients have fiducial markers implanted for image guidance.

Fiducial markers should be visible on CT and MRI imaging to allow image guidance and MRI/CT fusion. At least three fiducial markers will be placed under transrectal ultrasound guidance, using either transperineal or transrectal approach. Antibiotic cover with oral ciprofloxacin or equivalent and metronidazole per rectum, or equivalent, should be administered if fiducial placement is done transrectally. The operator will place seeds such that they are visible (and not superimposed) on orthogonal imaging (where used) and ideally are separated by 2 cm or more. Fiducials are usually placed as an outpatient procedure; at least three seeds must be usable for tracking translation and rotation during treatment. The use of one paired fiducial and two free fiducials (four in total) is recommended for CyberKnife SBRT treatment.

2.2 Hydrogel Spacers

Hydrogel spacers are permitted as part of the treatment for patients where this will not compromise target coverage or toxicity, and must not be used in patients with posterior T3a/T4 disease. In centres using hydrogel spacers, this must be used for patients randomised to either arm. A planning MRI must be used unless the spacer includes CT-visible contrast.

2.3 Planning CT and MRI scans

To allow fiducial stabilisation and resolution of swelling, planning studies are recommended to be imaged at least 7 days after fiducial placement.

CT scans will be taken for treatment planning. CT slices will be 1–1.5 mm. Patients who are randomised to pelvic node radiotherapy may be scanned with IV contrast to aid delineation as per department protocol. In addition, those with limited intra-abdominal fat may be considered for oral contrast administration. For CyberKnife SBRT scans will extend at least 15 cm above and below the level of the prostate, including the testes, so that these can be used as a blocking structure. For gantry-based SBRT, it is suggested that scans should extend from L2/L3 intervertebral space to 2 cm below ischial tuberosities.

It is strongly recommended that all patients undergo MRI imaging for radiotherapy planning purposes to determine the anatomical borders of the prostate and, if possible, the urethra. This is known to improve the accuracy of target contouring. The MRI will be fused to the treatment planning CT. It is recommended that MRI/CT fusion be done on implanted fiducials.

Bilateral hip prostheses, or any other implants/hardware that would introduce substantial CT artefacts and would make pelvic node planning more difficult, are excluded from the trial.

2.4 Patient Preparation and Positioning

2.4.1 Bowel Preparation

All patients should have their rectal diameter reviewed either using a short series scan localised in line with the prostate or on the completed CT planning scan. If the anterior/posterior diameter of the rectum is >4 cm at any level adjacent to the prostate the patient should be rescanned with additional bowel preparation. For patients who have a rectal AP diameter >4 cm despite re-scanning, acquire CBCT at fraction 1 to confirm similar rectal size. We suggest daily enemas for 2 days prior to, and on the day of, CT planning. We suggest patients should restart enemas 2 days prior to starting

radiotherapy. Patients are suggested to have an enema on each day of treatment unless they develop diarrhoea or proctitis.

2.4.2 Bladder Preparation

It is recommended that patients have a partially filled bladder (150-250 ml) during imaging and treatment delivery: patients should be asked to empty their bladder and then drink enough water (e.g. 325 ml) to ensure a reasonably filled bladder on the planning scan and before each fraction of radiotherapy. It is advised that the bladder should be filled to at least 150 ml to proceed with planning. However, this may not always be possible, and planning may proceed if agreed with the site Principal Investigator (PI).

2.4.3 Immobilisation

Patients will be scanned supine with arms across chest using an Alpha Cradle, vacbag or similar immobilization device, as needed. The use of knee and foot support is recommended, indexed to the treatment couch. Positioning and immobilisation should be as similar as possible during the planning MRI.

3 Organs at Risk and target volume definitions

3.1 Target Volume Definition

3.1.1 The Clinical Target Volume (CTV)

When using MRI-fusion images for voluming, it is acknowledged that the anatomy tends to be less consistent more superiorly, particularly at the level of the seminal vesicles. Therefore we recommend using MRI fusion for voluming the prostate and prostate/rectum interface, but where there is a discrepancy the CT anatomy should be prioritised. All other structures, other than urethra, should be outlined on CT.

3.1.2 CTV definition for PACE-NODES

There are three possible CTVs, defined as follows:

> CTVpsv = prostate plus <u>proximal 1 cm</u> of seminal vesicles (from insertion point in the sagittal plane, see Figure 1a) and any extraprostatic extension (periprostatic fat, seminal vesicle or base of bladder.) If T3b, if the extent of involved seminal vesicle is greater than the proximal 1 cm, the involved volume of the seminal vesicles will be included as CTVpsv.
>
> CTVsv = prostate plus <u>proximal 2 cm</u> of seminal vesicles (from insertion point in the sagittal plane, see Figure 1b). If preferred, and dose constraints allow, centres can elect to include the full length of SV within CTVsv, as per institutional practice, especially in cases with T3b disease.
>
> CTVn = pelvic lymph node volume (contour as per PIVOTALboost or PEARLS trial guidelines; note: extend up to L4/L5 vertebral interspace as for PEARLS).

3.1.3 Contouring Seminal Vesicles

This is the area of the protocol that can cause the most questions/QA discrepancies. The easiest way to contour the correct amount of seminal vesicle (SV) to include into the target volumes is to contour the prostate first, expand by 1 cm and by 2 cm, and to use these 1 cm / 2 cm ring structures as a guide for SV contouring (or automatically 'clip' the SVs at these borders using the planning system). This method is illustrated in Figure 1 and described below. As above, if preferred, centres can elect to include the full length of SV within CTVsv, as per institutional practice, especially in cases with T3b disease.

Note: If the anatomy is unusual and this method does not capture the anatomy you wish to treat, then it is permitted the outline the proximal 1 cm and 2 cm of seminal vesicles for CTVpsv and CTVsv freehand using the anatomy on the planning images. This should still effectively cover the same 'length' of SV from the point of insertion of the SV into the prostate.

If including the proximal 1 or 2 cm of SVs is felt to be clinically unsafe or inappropriate due to variant anatomy, then the proportion of SV included can be amended due to clinician discretion. If this occurs, please send a screenshot to the RTQA team as a record of why the volumes deviated from the protocol.

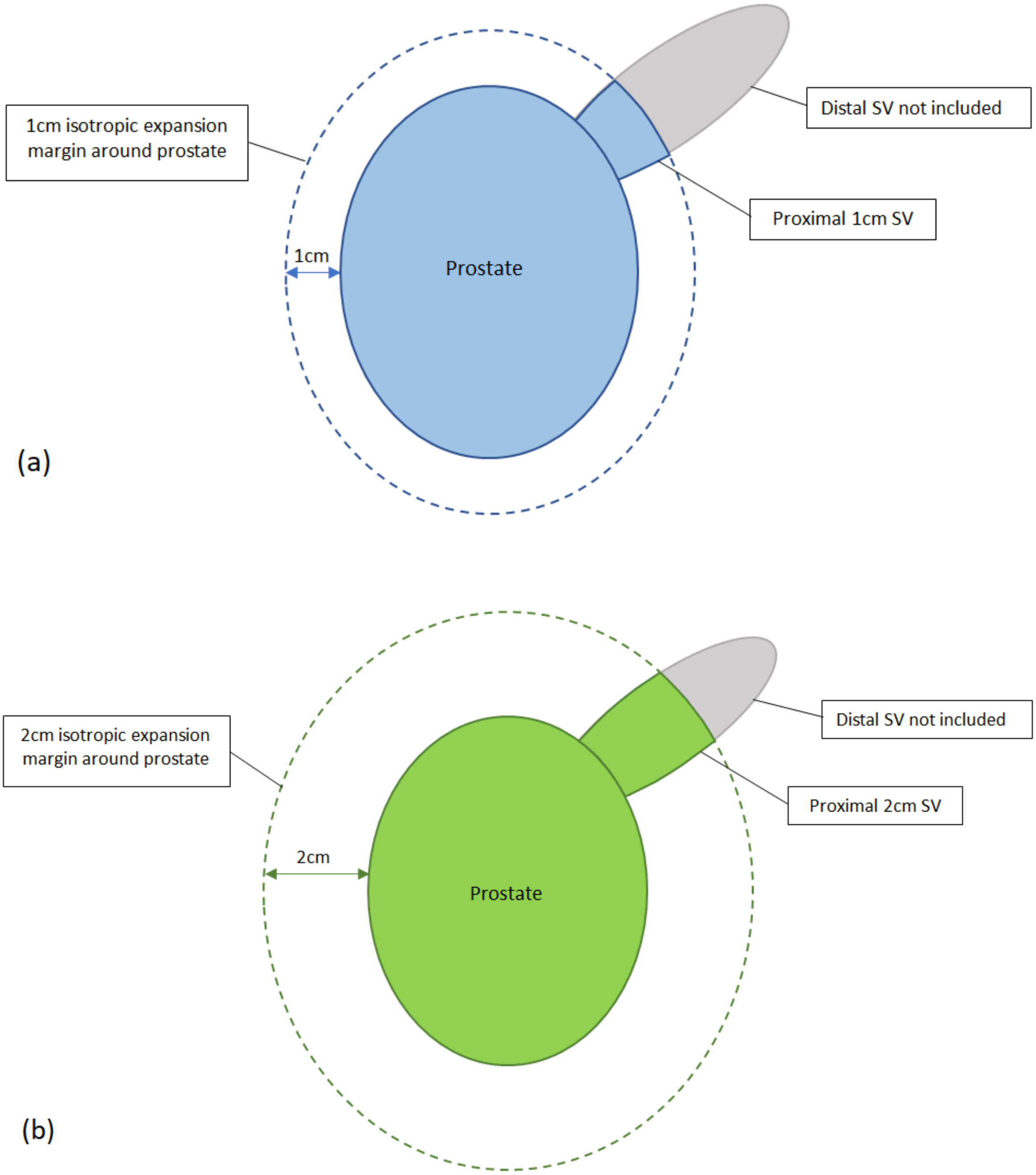

Figure 1. (a) Proximal 1 cm for CTVpsv (except for T3b patients when, if the extent of involved seminal vesicle is greater than the proximal 1 cm, the involved volume of the seminal vesicles will be included as CTVpsv) and (b) Proximal 2 cm (for CTVsv) of seminal vesicles. If preferred, centres can elect to

include the full length of SV within CTVsv, as per institutional practice, especially in cases with T3b disease.

First contour prostate (without SVs) and create 1 cm and 2 cm rings by isotropic expansion of the prostate (Figure 2). Note, if the expansion leaves a tiny sliver of SV on the top slice this can be deleted at the clinician's discretion.

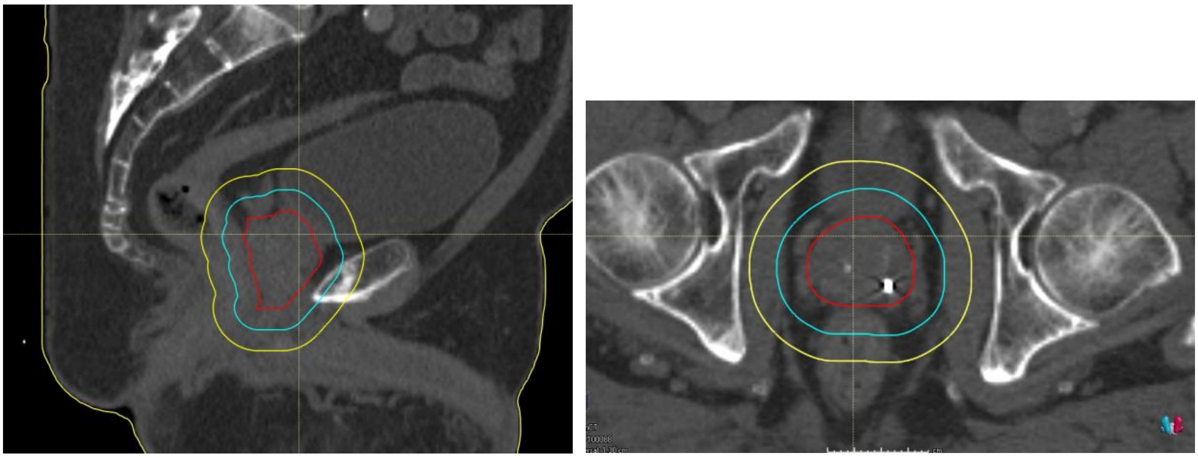

Figure 2. Sagittal (left) and axial (right) images of prostate (red) expanded by 1 cm (cyan) and 2 cm (yellow) to create isotropic rings.

After contouring the whole SV, the proximal 1 cm of SV is added to the prostate volume to form CTVpsv. The SV at 1-2 cm is coloured green, and the entire proximal 2 cm of SV will be added to the prostate volume to form CTVsv. See Figure 3.

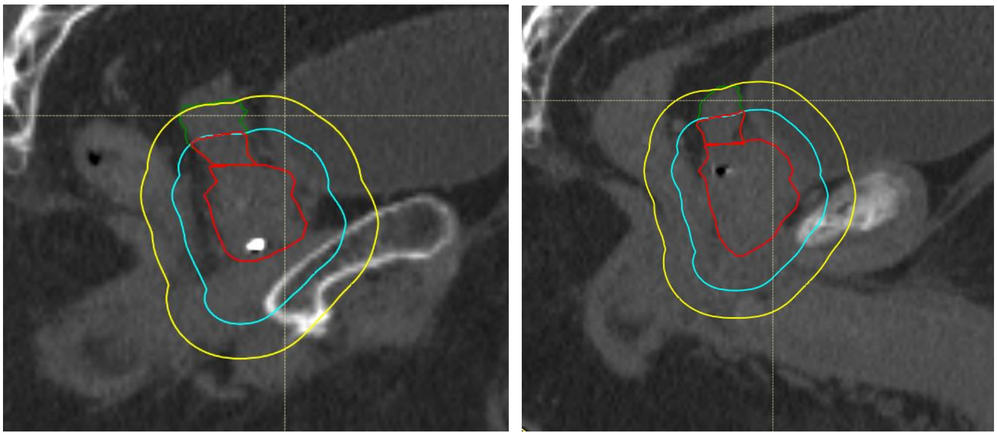

Figure 3. Sagittal images showing proximal 1 cm of SV (red) and the SV at 1-2 cm (green).

3.2 Organs at Risk

The following organs at risk (OAR) will be contoured. These are given in reducing order of priority for planning constraints.

<u>Bowel</u>: The individual bowel loops visible on relevant levels of the planning scan will be outlined and will be used for the 'bowel' dose-volume constraint. The outlining will include the small bowel, the large bowel and the sigmoid colon, down to the level of the recto-sigmoid junction. The superior

extent of outlining should be 2 cm beyond the superior extent of CTVpsv or CTVn as appropriate. Ensure small bowel in the lower pelvis caudal to the recto-sigmoid junction is included.

Rectum: defined as a solid structure, including the lumen and rectal wall, extending from (and including) the anus to the rectosigmoid junction. The rectum should be contoured to the outer boundary of the external rectal wall, including rectal contents. Outlining should extend from the bottom of the ischial tuberosities or the anal margin (whichever is inferior) to the recto-sigmoid junction approximately at the level of the S3 vertebral body. The rectosigmoid flexure is best visualized on sagittal viewing planes.

Bladder: defined as a solid structure including the bladder wall and lumen.

Urethra if visible (best seen on MR, especially T2 weighted sagittal images): the prostatic urethra is defined as the lumen/mucosal interface, extending from bladder neck to the membranous urethra. If a planning MRI is used it is mandatory to contour urethra.

Penile bulb: the portion of the bulbous spongiosum that lies inferior to the urogenital diaphragm. The penile bulb is most easily seen on the planning MRI.

Femoral heads: Each femoral head should be contoured separately. The femoral heads are outlined to the bottom of the curvature of their heads (femoral necks are not included). Contour on bone windows.

Testes: For CyberKnife SBRT, beams should not be allowed to traverse the testes due to the effects on hormone production and subsequent confusion of biochemical outcomes [1]. The bilateral testes should therefore be used as a ‘blocking structure’.

3.3 Structure Naming Convention

As an NCRN radiotherapy trial, the PACE-NODES study uses a standardised naming convention [2]. This will avoid ambiguity and facilitate analysis of radiotherapy plan data. This convention is detailed in Table 1.

**Volume**	**Naming convention (includes target dose in cGy for target volumes)**	
	**P-SBRT**	**PPN-SBRT**
Clinical target volume: Prostate, seminal vesicles +/- nodes	CTVpsv, CTVsv	CTVpsv, CTVsv and CTVn
SBRT treatment volumes		
Clinical target volume: prostate +/- seminal vesicles (receives 40 Gy)	CTVpsv_4000	CTVpsv_4000
Planning target volume: prostate +/- seminal vesicles (receives 36.25 Gy) +/- nodes (receives 25 Gy)	PTVpsv_3625 and PTVsv_3000	PTVpsv_3625 and PTVsv_3000 and PTVn_2500
Organs at risk	***Approved PACE-NODES nomenclature***	
Rectum	Rectum	
Bladder	Bladder	
Urethra	Urethra	
Left femoral head	FemurHead_L	

Right femoral head	FemurHead_R
Penile bulb	PenileBulb
Bowel	Bowel

Table 1: Structure naming convention for PACE-NODES

4 Margins for Planning Target Volumes

Planning target volumes (PTV) will be outlined and reported in line with ICRU 83 “Prescribing, recording and reporting photon-beam intensity modulated radiotherapy (IMRT)” [3] and ICRU 91 “Prescribing, recording and reporting of stereotactic treatments with small photon beams” [4] where relevant.

4.1 PTV Margins

Preferred PACE-NODES margins for SBRT, assuming fiducials or equivalent, such as MRL, are used:

PTVsv_3000 = CTVsv + 5 mm

PTVpsv_3625 = CTVpsv + 5 mm / 3 mm posteriorly

(Additionally CTVpsv_4000 = CTVpsv with no margin)

Permissible range for margins if above convention is not used

PTVsv_3000 = CTVsv + 6 mm

PTVpsv_3625 = CTVpsv + 5 mm / 4-5 mm posteriorly

For patients in the PPN-SBRT arm:

PTVn_2500 = CTVn+ 5 mm

5 External Beam Radiotherapy Planning Guidelines

5.1 Radiotherapy Technique

This trial simultaneously treats multiple dose-level PTV structures in a single phase. Therefore, an IMRT or VMAT planning technique must be used to obtain the prescription doses for adjacent PTVs.

All radiotherapy techniques are to be approved in advance by the Chief Investigator and trial QA team.

5.2 Prescribed Dose and Fractionation

The prescription dose will be 36.25 Gy given in 5 fractions over 1-2 weeks (i.e. alternate days). The prescription dose of 36.25 Gy shall be the dose to PTVpsv_3625.

Structure	Contains / derived from	Dose (Gy)
PTVpsv_3625	Prostate+proximal 1 cm of SV (CTVpsv)*	36.25 (40 to CTV)
PTVsv_3000	Prostate+proximal 2 cm of SV (CTVsv)^	30.0
Note: there is an additional target volume CTVpsv_4000 (CTVpsv with no margin) which receives 40 Gy		

Table 2. Dose to PTVs in PACE-NODES - P-SBRT

Structure	Contains / derived from	Dose (Gy)
PTVpsv_3625	Prostate+proximal 1 cm of SV (CTVpsv)*	36.25 (40 to CTV)
PTVsv_3000	Prostate+proximal 2 cm of SV (CTVsv)^	30.0
PTVn_2500	Pelvic lymph nodes	25.0
Note: there is an additional target volume CTVpsv_4000 (CTVpsv with no margin) which receives 40 Gy		

Table 3. Dose to PTVs in PACE-NODES - PPN-SBRT

* for T3b disease, extend CTVpsv to the **most distal extent** of the involved SV or proximal 1 cm, whichever is furthest

^ If preferred, and where dose constraints allow, centres can elect to include the full length of SV within CTVsv, as per institutional practice, especially in cases with T3b disease.

5.3 Definition of PTVs for Dose Reporting with their Dose Constraints

For SBRT planning, the prescription isodose (to PTVpsv) shall be 75-85% of Dmax, and aim for D98% ≥ 34.4 Gy. This may need to be relaxed to achieve the mandatory rectal constraint.

5.3.1 P-SBRT

Planning target volume	Dose to PTV
PTVpsv_3625	V36.25 Gy ≥ 95%; D98% ≥ 34.4 Gy
CTVpsv_4000	A secondary dose of 40 Gy should be delivered to the CTVpsv such that the CTV V40Gy ≥ 95%
PTVsv_3000	Dose to PTVsv_3000 will be prescribed to a ring structure excluding PTVpsv_3625 (i.e. PTVsv_3000 minus PTVpsv_3625), for which the following dose objective will be met: V30 Gy ≥ 95%

5.3.2 PPN-SBRT

Planning target volume	Dose to PTV
PTVpsv_3625	V36.25 Gy ≥ 95%; D98% ≥ 34.4 Gy
CTVpsv_4000	A secondary dose of 40 Gy should be delivered to the CTVpsv such that the CTV V40Gy ≥ 95%
PTVsv_3000	Dose to PTVsv_3000 will be prescribed to a ring structure excluding PTVpsv_3625 (i.e. PTVsv_3000 minus PTVpsv_3625), for which the following dose objective will be met: V30 Gy ≥ 95%
PTVn_2500 *	Dose to PTVn_2500 will be reported to a cropped structure out_PTVn = PTVn_2500 - (PTVsv_3000 + 0.5 cm), for which the following dose objectives will be met: D98% ≥ 23.75 Gy (95%); D50% (median) = 25 Gy ± 2%; D2% ≤ 26.75 Gy

5.4 Normal Tissue Dose Constraints for Organs at Risk

Organ at risk	Dose volume constraints		
	Dose (Gy)	Maximum volume (% or cc)	
		Mandatory	Optimal
Rectum	18.1	50%	
	29	20%	
	36	2cc	1cc
Bladder	18.1	40%	
	37	10cc	5cc
Femoral heads †	14.5	-	5%
Bowel (P-SBRT)	18.1	5cc	
	30	1cc	
Penile bulb	29.5	-	50%
Urethra (if visualised)	42	-	50%
	45	-	<0.001cc
Testicular	Blocking structure		

† When reporting femoral head V40Gy, report the larger calculated volume only

Table 4. OAR dose constraints for P-SBRT

Organ at risk	Dose volume constraints		
	Dose (Gy)	Maximum volume (% or cc)	
		Mandatory	Optimal
Rectum	18.1	50%	
	29	20%	
	36	2cc	1cc
Bladder	18.1	40%	
	37	10cc	5cc
Femoral heads †	14.5	-	5%
Bowel (PPN-SBRT)	18.1	150cc	100cc
	25	20cc	10cc
	30	1cc	
Penile bulb	29.5	-	50%
Urethra (if visualised)	42	-	50%
	45	-	<0.001cc
Testicular	Blocking structure		

† When reporting femoral head V40Gy, report the larger calculated volume only

Table 5. OAR dose constraints for PPN-SBRT

5.5 Dose-Volume Variations

Structure	Minor variation	Major variation
Target volumes		
CTVpsv_4000	V40Gy = 90-94.9%	V40Gy < 90%
PTVpsv_3625	V36.25Gy = 90-94.9%	V36.25Gy < 90%
PTVn_2500	V23.75Gy = 90-97.9%	V23.75Gy < 90%

Table 6. Minor and Major variations for SBRT dose reporting

Investigators shall attempt to keep normal tissue doses and prescription coverage as close to “per protocol” specifications as possible. If all the above “per protocol” dose-volume criteria cannot be met on a given patient, then target prescriptions may be relaxed to the “minor variation” range as follows: one minor variation in one of the target volumes in Table 6 (i.e. PTVpsv V36.25Gy = 90-94.9% or CTVpsv V40Gy = 90-94.9% or PTVn V23.75Gy = 90-97.9%) is allowed; two or more minor variations or one major variation are allowed only with the consent of the local PI. Variations can be discussed with the RTQA team or PACE-NODES clinical leads if required.

If an adequate plan cannot be achieved within these constraints, please review the contours and discuss with the RTQA team and/or the CI. All variations shall be noted.

6 Radiotherapy Delivery

6.1 Treatment Scheduling

It is highly recommended that radiotherapy start within 8 weeks of randomisation, but it must start within 16 weeks.

Treatment will be given in a single phase on alternate weekdays, over no more than 14 days; longer planned treatment durations are to be discussed with a Clinical Leads for approval. Overall treatment duration will be recorded.

Where treatment fractions are missed, these can be added on to the end of treatment on alternate days for prostate and pelvic nodes SBRT or on consecutive or alternate days for prostate-only SBRT.

6.2 Treatment Verification

All patients will have daily image-guided radiotherapy to the prostate, and it is strongly recommended that this is done with fiducial guidance and volumetric imaging. For gantry-based SBRT using cone beam CT (CBCT) without fiducials, centres must demonstrate that they are experienced in soft tissue matching to the prostate. 2D imaging is only permissible if prostate fiducial markers are used. In addition, volumetric imaging pre-treatment is encouraged to rule out any significant changes in rectal position or prostate deformation.

It is important to ensure that patients follow the bladder/bowel preparation instructions they used at the planning CT scan appointment. For patients with rectal diameter >4 cm at CT, day 1 CBCT should confirm rectal size is comparable. Where bladder filling is insufficient at planning CT, i.e. <150 ml, adjustment to drinking instructions should be made where relevant, with review of bladder volume assessed by CBCT.

For all patients, it is mandatory that couch shifts are applied online for all displacements. Please discuss with the RTQA team if this is not possible.

Time from imaging acquisition completion (i.e. when images are available to review) to beam on should be less than 2 minutes for P-SBRT and less than 4 minutes for PPN-SBRT to reduce the risk of prostate motion while allowing time for the nodal match. If it takes longer, consider re-imaging the patient at your discretion, e.g. if the anatomy appears unstable. MR-guided radiotherapy is permitted, with or without daily adaptive re-planning.

Specific IGRT considerations for prostate only, prostate and pelvic nodes and intra-fraction motion control are discussed below.

Prostate only IGRT

Volumetric imaging is used to daily match either the implanted markers or, if not possible and experience allows, soft tissue match to the prostate. 2D imaging is only permissible if prostate fiducial markers are used (a minimum of 2 fiducial markers is allowed but 3 fiducials are recommended). If <2 fiducial markers are visible, additional fiducials can be placed and the patient re-planned. Otherwise, CBCT match to soft tissue would be required (using the larger CBCT PTV margins), experience permitting.

If, at fraction 1, any shift value is greater than 10 mm, check the bone match, rectal and bladder filling and, if correctable, re-position and re-image. If the set-up error cannot be effectively resolved, do not commence treatment until the images have been reviewed by a locally assigned responsible person (e.g. PI, senior radiographer, IGRT lead, physicist). In rare occasions, a re-scan and replan may be required.

If a significant shift is required (>5 mm), the patient should be re-imaged after that shift is applied. It is recommended that, if the initial image was 2D, the second image be volumetric (3D) to allow visualisation of OAR deformation. If shifts >5 mm occur on more than one occasion, arrange an offline review by a locally assigned responsible person (e.g. PI, senior radiographer, IGRT lead, physicist).

Where the ability exists, rotational corrections may be applied. Correction of prostate-only rotations may not have a significant impact on prostate coverage.

Prostate and Pelvic Node IGRT

Volumetric imaging is used to match bony anatomy and implanted markers or, if not possible and experience allows, bony anatomy and soft tissue match to the prostate.

Initial match to bones with region of interest (ROI) to cover prostate and as much nodal volume as practical. Perform bone match and record shifts. Rematch to the prostate only using the implanted markers or, if not possible, soft tissue match to prostate. If the difference between the prostate and nodal (bone) match is ≤5 mm, apply the shift (based on prostate alignment) and continue with treatment.

If the **difference between bone and prostate isocentre shifts is >5 mm**:
Review the image for set-up issues influencing the mismatch between bone and prostate soft tissue. Review rectal and/or bladder filling. If significantly different, instruct patient as appropriate. For example, if the patient’s rectal diameter is large and pushing the prostate anteriorly or the patient’s bladder is full and pushing the prostate inferiorly. If the identified factor is amenable to improvement, apply the appropriate intervention, e.g. patient to evacuate rectum or empty bladder and repeat setup.
Do vessels within the CTVn lie outside PTVn by more than 1 mm over at least 3 slices? If so, try to determine cause of difference. Did the patient tense their buttocks at planning CT and now relaxed?

If **after re-setup the deviation is >5 mm but ≤10mm,** and all efforts to correct deviation have been made, then accept deviation prioritising the prostate coverage. If this occurs on another fraction, flag for offline review with a locally assigned responsible person (e.g. PI, senior radiographer, IGRT lead, physicist) for clinical and dosimetric review.

If **after re-setup the deviation is >10 mm, do not treat** and arrange offline review with a locally assigned responsible person (e.g. PI, senior radiographer, IGRT lead, physicist) for clinical and dosimetric review.

In rare occasions, a re-scan and replan may be required.

Where the ability exists, rotational corrections may be made but the nodal PTV coverage should be reviewed prior to treatment.

Intra-fraction Motion Control

For SBRT using CyberKnife, patients will have fiducial-based intra-fraction motion corrected during treatment.

For centres using Elekta Clarity ultrasound monitoring, prostate motion will be monitored continually and treatment paused (and position corrected) if prostate displacement exceeds 5 mm. For Clarity, a 5 mm tolerance with a 5 second threshold is recommended.

For SBRT with gantry-based systems, it is anticipated that the majority of centres will use an arc-based IMRT technique, with or without flattening filter-free delivery. Flattening filter-free delivery should have a beam on time of under 3 minutes, in which case intrafraction motion control is not mandated. Where beam-on time exceeds 3 minutes, re-imaging should occur between beams/arcs (or at approximately 3-4 minute intervals).

7 Documentation on Completion of Radiotherapy

Radiotherapy plan data will be collected (in DICOM format by electronic transfer – see section 8.5 - plus Plan Assessment Form in Excel format) for all patients having radiotherapy within the trial. This data will be stored on a secure server by the sponsor.

8 Radiotherapy Quality Assurance

8.1 Radiotherapy Quality Assurance Overview

Radiotherapy quality assurance (RTQA) includes pre-trial and on-trial components. RTQA will be streamlined where feasible with other prostate trials, e.g., PIVOTALboost, PEARLS, PACE – see section 8.2.3 for details.

RTQA documentation and data can be downloaded from the RTTQA website:

http://www.rttrialsqa.org.uk

Please send all completed RTQA to the PACE-NODES RTQA contact at: pace-nodes.rtqa@nhs.net

Pre-trial QA includes:

- Benchmark outlining case
- Benchmark planning case
- Facility questionnaire

On-trial QA includes:

- Prospective and/or retrospective case reviews

- Dosimetry site visit (subject to prior RTTQA dosimetry accreditation)

All outlining should be either performed by, or reviewed and approved by, the PI at the centre who has been through the pre-trial outlining QA. Since this is a clinical trial and the patient numbers may not be excessive, we hope this approach will be acceptable. However, where this is not feasible, the PI should review and approve clinical outlines for the first cohort of PACE-NODES patients recruited by each additional clinician at that centre to include a minimum of 3 pelvic lymph node contours, after which (assuming these are satisfactory) they are also approved for PACE-NODES.

Please notify the RTQA team of any additional clinicians who have been locally approved in this way. A form is available from the RTQA team to facilitate this.

Should the PI leave and be replaced, the new PI should complete the PACE-NODES benchmark outlining QA.

8.2 Pre-trial QA

8.2.1 Facility Questionnaire

The Prostate Facility Questionnaire (FQ) collects information about the radiotherapy equipment, techniques and procedures used by a centre for the trial.

The questionnaire will be sent to each site by the RTQA team as it may be pre-filled with information provided for previous trials. If the FQ has been pre-filled, please update where necessary to reflect procedures and equipment for the PACE-NODES trial and re-submit.

8.2.2 Benchmark Cases

An outlining and a planning benchmark case will be completed by all trial centres. QA may be streamlined for those centres who have completed RTQA accreditation for other prostate trials (see section 8.2.3).

8.2.2.1 Downloading data from the RTTQA website

Download the benchmark case DICOM datasets from the RTTQA website:

http://www.rttrialsqa.org.uk

- Log in
- Select ‘Downloads’ from the menu bar
- Open ‘Top Level Folders’ (see below) and select the ‘PACE-NODES’ folder
- Select files and choose ‘Download’ from the ‘Action’ column

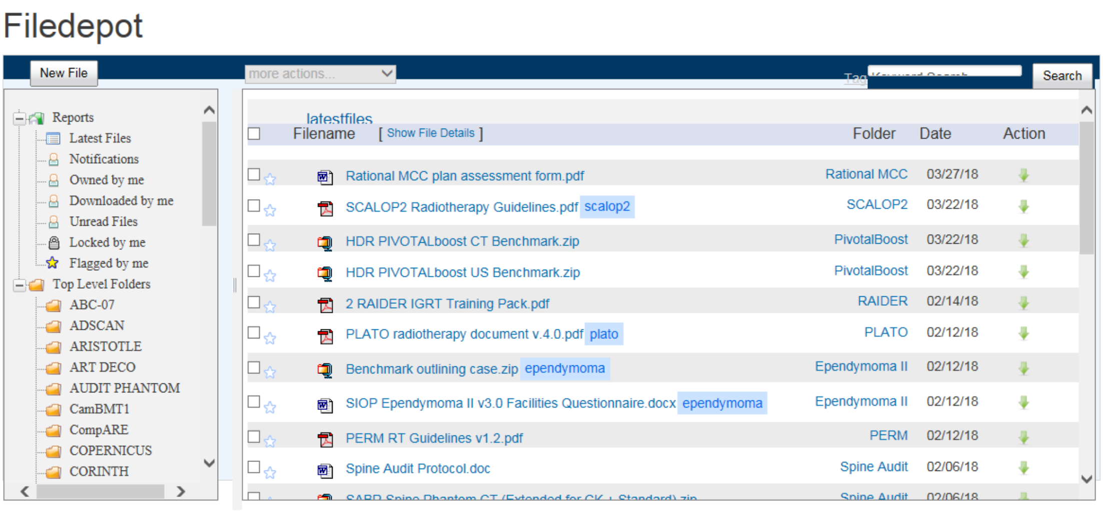

8.2.2.2 Outlining Benchmark Case

All centres wishing to participate in the PACE-NODES trial will need to complete a contouring exercise for prostate and pelvic nodes unless both the site and the site PI have completed the outlining benchmark case for PIVOTALboost or PEARLS. The planning MRI, planning CT and a PDF file containing the clinical history (file PACE_NODES_outlining_benchmark.zip) should be downloaded from the RTTQA website. Please import the DICOM data into your own outlining or treatment planning system (TPS). It is not mandatory to use the planning MRI, but if you wish to do so you will need to register it to the planning CT.

Refer to outlining instructions in Section 3, supplemented by the PEARLS Pelvic Lymph Node Contouring document (Section 3 only). Please use the trial structure naming convention.

Once outlines have been created, reviewed and accepted by the local PI, please export and return the DICOM CT and Structure data to the RTQA team using RTTQA data transfer, see section 8.5.

8.2.2.3 Planning Benchmark Case

All sites must complete and submit the PACE-NODES planning benchmark case for the prostate and pelvic lymph nodes arm. The CT images and pre-outlined structure set are available for download from the RTTQA website.

Please import the CT images and structure set into your own TPS. The CT has been delineated by the CI with the following structures, which should not be edited. No additional structures, e.g. for PTV/OAR overlaps, have been created; the individual centre should create these as needed.

- Target volumes: CTVpsv, CTVsv, CTVn
- OARs: Rectum, Bladder, Bowel, FemurHead_L and _R, PenileBulb

You will need to create the PTVs.

This patient should be planned as a prostate and pelvic lymph nodes case.

Please complete the PACE-NODES Plan Assessment form (PAF) and provide a copy of the TPS treatment planning report (for beam configuration details).

Data Export: Once the benchmark plan has been created, reviewed and accepted by the local PI, the export of the CT images, dose matrix, RT plan and structure set in DICOM format should be returned

to the RTQA team (see section 8.5). Avoid re-anonymising as this causes problems and may delay your review.

Note that RTQA approval can be obtained when the benchmark cases have been approved. However, you cannot recruit your first patient (be "activated") until you have returned your Facility Questionnaire and had it approved.

8.2.3 Streamlining QA

RTQA will be streamlined using RTQA submitted for other prostate trials where feasible:

- The Facility Questionnaire must be completed by all trial centres, but will be pre-filled by the RTQA team if you have completed the Facility Questionnaire for another prostate trial.
- The outlining benchmark case will be streamlined if BOTH the PI and the centre have completed outlining RTQA for another prostate trial as follows:
    - PIVOTALboost or PEARLS – Outlining benchmark case is not required
    - PACE – Contour only the pelvic node structures (Vessel, CTVn) and Bowel
- All trial centres must complete the planning benchmark case.

8.3 On-Trial QA

8.3.1 Patient Case Reviews

The outlining and treatment planning for the first cohort of patients recruited by each trial centre will be subject to review by the RTQA team. This may be a prospective (i.e. pre-treatment) or timely retrospective review, to be advised by the RTQA team on a case-by-case basis. Additional reviews may be requested by the RTQA team as required.

To ensure a short response time for prospective reviews please notify the RTQA team when a patient has been identified, and please allow 2 weeks between submitting data and the RT treatment start date. Please send outlining for review in advance of RT treatment planning where possible to expedite the review. Failure to give the QA team sufficient notice of a case may result in delays in the case being reviewed. Should it not be possible to complete a review prior to the planned treatment start date, it is the PI's responsibility to decide whether to start treatment as planned (prepared to re-plan for remaining treatment fractions if necessary) or to delay treatment start until review is complete.

For outlining reviews please send:

- Planning CT images
- Planning MR images and DICOM registration object(s) (if used)
- DICOM structure set

For planning reviews please send:

- Planning CT images
- DICOM structure set
- DICOM dose matrix
- DICOM plan file
- Completed PAF
- Copy of TPS treatment plan report (for beam configuration details)
- Patient-specific QA results (measured or calculated); may be sent following the review

See section 8.5 for data export instructions.

Note the following:

1. All retrospective reviews are "timely": data to be submitted within a week of treatment start and review to be completed before another trial patient is treated.
2. The RTQA team will be in touch if a patient is recruited who needs a prospective review.

8.3.2 Dosimetry Audit

All sites are required to have a recent dosimetry site visit by the RTTQA group. Sites which do not have this will be contacted individually by the RTTQA team to arrange an audit.

8.4 Ongoing Data Collection

Radiotherapy plan data will be collected (in DICOM format by electronic transfer) for all patients having radiotherapy within the trial. These data will be stored on a secure server by the sponsor. All patient data must be anonymised before transfer, and should be re-identified with the trial number. The Plan Assessment Form should also be sent.

Plans should ideally be submitted once they have been approved by the PI and had an independent check. Data associated with any re-plans during radiotherapy treatment should also be submitted.

Please send the following data:

- Planning CT images
- Planning MR images and DICOM registration object(s) (if used)
- DICOM structure set (ensure trial naming convention has been followed)
- DICOM dose matrix
- DICOM plan file
- Completed Plan Assessment Form (original Excel format)

8.5 DICOM Data Export

DICOM data should be transferred to the RTTQA group via their central secure transfer service. Anonymised and encrypted data will pass through a firewall to a host server located in a secure NHS environment to which access is restricted to authorised users.

All NHS radiotherapy centres have a centre specific link and unique password to access the service – contact the RTQA team if you do not have this.

Instructions for use:

1. Data must be anonymised at source and should be encrypted using 7zip/WinZip or equivalent
2. All files for a single patient must be zipped into one file
3. Files must be labelled with the Trial Name and Trial ID, e.g. *example_trial_ID*
4. Follow unique centre link to the service

RTTQA
Radiotherapy Trials Quality Assurance

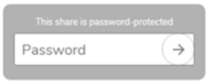

5. Insert unique centre password

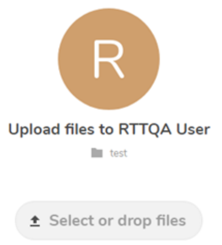

6. Upload files
7. Email RTQA contact at pace-nodes.rtqa@nhs.net to confirm data uploaded and share password to unzip data

9 References

[1] King, C.R., A. Lo, and D.S. Kapp, Testicular dose from prostate CyberKnife: a cautionary note. Int J Radiat Oncol Biol Phys, 2009. 73(2): p. 636-7; author reply 637.

[2] Mir R, Kelly SM, Xiao Y et al. Organ at risk delineation for radiation therapy clinical trials: Global Harmonization Group consensus guidelines. Radiother Oncol, 2020. 150: p. 30-39. https://doi.org/10.1016/j.radonc.2020.05.038

[3] ICRU (International Commission on Radiation Units and Measurements), Prescribing, recording, and reporting photon-beam IMRT. Report No. 83, Journal of the ICRU Vol. 10, Oxford University Press, Oxford, U. K., 2010.

[4] ICRU, Prescribing, recording and reporting of stereotactic treatments with small photon beams. Report No. 91.
