# Supplementary material for: PACE-NODES: A phase III randomised trial of 5 fraction prostate stereotactic body radiotherapy (SBRT) versus 5 fraction prostate and pelvic nodal SBRT
Source: Clin Transl Radiat Oncol. 2026 Feb 11;58:101126. doi: 10.1016/j.ctro.2026.101126 (PMC12925429; doi:10.1016/j.ctro.2026.101126)
Supplement: Supplementary Data 3 [file mmc3.pdf]

Appendix C: PACE-NODES Trial Management Group (TMG) and Protocol Development Group (PDG) membership

TMG members:

Nicholas van As	Chief Investigator	The Royal Marsden NHS Foundation Trust
Emma Hall	Director	ICR-CTSU, The Institute of Cancer Research, London
Angela Pathmanathan	Clinical Lead	The Royal Marsden NHS Foundation Trust
Suneil Jain	Clinical Lead	Queen's University, Belfast
Ananya Choudhury	Chair and Honorary Consultant in Clinical Oncology	University of Manchester
John Staffurth	Professor in Clinical Oncology	Cardiff University
Alison Tree	Consultant Clinical Oncologist	The Royal Marsden NHS Foundation Trust
Peter Hoskin	Consultant Clinical Oncologist	University of Manchester
Conor McGarry	Medical Physicist	Belfast Health & Social Care Trust
Elizabeth Miles	National RTTQA Group Lead	Mount Vernon Cancer Centre
Olivia Naismith	Radiotherapy Trials Physicist	The Royal Marsden NHS Foundation Trust
Yee Pei Song	Consultant Clinical Oncologist	The Christie NHS Foundation Trust
Isabel Syndikus	Consultant Clinical Oncologist	The Clatterbridge Cancer Centre
Vedang Murthy	Consultant Clinical Oncologist	Tata Memorial Centre, Mumbai
Tim Ward	Patient Representative	
Ken McBride	Patient Representative	
Stephanie Burnett	Clinical Trials Programme Manager	ICR-CTSU, The Institute of Cancer Research, London
Fay Cafferty	Lead Statistician	ICR-CTSU, The Institute of Cancer Research, London
Stephanie Brown	Senior Trial Manager	ICR-CTSU, The Institute of Cancer Research, London
Monisha Dewan	Trial Statistician	ICR-CTSU, The Institute of Cancer Research, London

Protocol development group:

Nicholas van As	Chief Investigator	The Royal Marsden NHS Foundation Trust
Emma Hall	Director	ICR-CTSU, The Institute of Cancer Research, London
Angela Pathmanathan	Clinical Lead	The Royal Marsden NHS Foundation Trust
Suneil Jain	Clinical Lead	Queen's University, Belfast
Ananya Choudhury	Chair and Honorary Consultant in Clinical Oncology	University of Manchester
John Staffurth	Professor in Clinical Oncology	Cardiff University
Alison Tree	Consultant Clinical Oncologist	The Royal Marsden NHS Foundation Trust

Julia Murray	Consultant Clinical Oncologist	The Royal Marsden NHS Foundation Trust
Peter Hoskin	Consultant Clinical Oncologist	University of Manchester
Conor McGarry	Medical Physicist	Belfast Health & Social Care Trust
Elizabeth Miles	National RTTQA Group Lead	Mount Vernon Cancer Centre
Olivia Naismith	Radiotherapy Trials Physicist	The Royal Marsden NHS Foundation Trust
Yee Pei Song	Consultant Clinical Oncologist	The Christie NHS Foundation Trust
Isabel Syndikus	Consultant Clinical Oncologist	The Clatterbridge Cancer Centre
Tim Ward	Patient Representative	
Stephanie Burnett	Clinical Trials Programme Manager	ICR-CTSU, The Institute of Cancer Research, London
Fay Cafferty	Lead Statistician	ICR-CTSU, The Institute of Cancer Research, London
